# Chromatin accessibility regulates age-dependent nuclear mechanotransduction

**DOI:** 10.1073/pnas.2522217123

**Published:** 2026-03-26

**Authors:** Yawen Liao, Luezhen Yuan, Trinadha Rao Sornapudi, Max Land, Rajshikhar Gupta, G. V. Shivashankar

**Affiliations:** ^a^Laboratory of Multiscale Bioimaging, Paul Scherrer Institute, Villigen 5232, Switzerland; ^b^Department of Health Sciences and Technology, ETH Zurich, Zurich 8092, Switzerland; ^c^Broad Institute of Massachusetts Institute of Technology and Harvard, Cambridge, MA 02142; ^d^Massachusetts Institute of Technology, Cambridge, MA 02139

**Keywords:** cellular aging, TGF-β signaling, mechanotransduction, 3D chromatin, gene expression

## Abstract

The integration of environmental cues into cellular programs is crucial for cell function. Yet, how this integration is modulated due to cellular aging remains unclear. We propose that the 3D chromatin organization filters these signals and investigated how age-related chromatin changes in human dermal fibroblasts affect responses to mechanical tension and TGF-β. Young fibroblasts exhibited synergistic gene expression enhancement in response to combined stimuli, a response that was markedly blunted or divergent in aged cells. These distinct outcomes correlated with significant age-related differences in chromatin accessibility. We identified the AP-1 complex and other transcription factors with age-specific activity as pivotal in remodeling chromatin and orchestrating these divergent mechanochemical responses during cellular aging. We validated that disrupting AP-1 activity inhibits fibroblast activation by preventing JUNB recruitment to the transcription machinery. Our findings establish chromatin as a key integrator of mechanochemical signals and characterize the age-related alterations to this integration that modify the cellular responsiveness of aged cells, highlighting AP-1 and its network as potential therapeutic targets against age-related decline.

## Significance

Although cells continuously integrate diverse environmental cues, the mechanisms by which aging reprograms mechanical and biochemical signaling remain poorly understood. We demonstrate that aged fibroblasts lose the capacity to mount synergistic responses to simultaneous mechanical and TGF-β stimulation, driven by age-dependent alterations in chromatin accessibility. These findings establish a paradigm in which 3D genome architecture functions as a critical regulator of mechanochemical information, and its age-related dysregulation reprograms cellular responsiveness. We further identify the AP-1 network as a master regulator of this process.

A major complication to the study of biology is the vast heterogeneity that exists on the cellular level. In fact, it is a well-recognized biological observation that even cells of the same type can exhibit distinct and varied responses when subjected to an identical stimulation (1, 2). This inherent cell-to-cell variability in responsiveness is not simply random noise but a fundamental characteristic of biological systems, influencing processes from development to disease (3, 4). However, the precise molecular mechanisms that govern this fundamental behavior and dictate such differential outcomes remain incompletely understood, presenting a significant challenge in comprehending cellular decision-making and function.

In the context of aging, this cellular heterogeneity often becomes more pronounced, with aging cells frequently displaying distinct gene expression responses to the same signals compared to their younger counterparts (5). Numerous studies have indicated that the aging process can lead to functional declines in critical cellular signaling pathways (6–9). In part, this is because cellular aging is strongly associated with substantial alterations in chromatin, encompassing changes to local chromatin structures (10, 11), shifts in higher-order chromatin organization (12, 13), and widespread epigenetic modifications (14, 15). These age-related epigenetic changes and remodeling of chromatin architecture are increasingly implicated as key contributors to the altered transcriptional landscapes and functional responsiveness observed in aging cells (16, 17).

In concert with these genomic shifts in aging, our foundational work has demonstrated that cellular gene expression programs are significantly influenced by distinct geometric constraints, even within the same cell type (18). This was further corroborated by findings that TNFα elicited disparate gene expression profiles in cells on different geometric substrates, underscoring a mechanical state dependency in cytokine signaling responses (19). This is true not only for cells in different synthetic mechanical states but also for cells in native environments, as cells in different locations throughout the ECM experience different mechanical environments. We explored whether these intrinsic cellular mechanical state differences are reflected in geometry-sensitive nuclear morphology and chromatin organization, and found that they modulate fibroblast activation in 3D environments (20). Finally, we expanded these investigations into the heterogeneity of cell responses to include pathological states in the tumor microenvironment (21), and showed that mechanical stress synergizes with cytokine signaling to augment inflammation and induce chromatin reorganization (22). However, the fundamental mechanics governing these observed phenomena require further clarification, particularly in the context of cellular aging.

Addressing this gap, we hypothesize that the 3D organization of chromatin acts as a crucial signaling filter, thereby dictating the specificity of these differential outcomes. We propose that this structural gatekeeping at the chromatin level—for instance, by modulating the accessibility of key gene regulatory elements to signaling-activated transcription factors (TFs)—could elucidate how broadly acting environmental cues are translated into precise and context-specific cellular programs. This framework is particularly relevant as 3D chromatin architecture is known to undergo substantial remodeling during cellular aging. Therefore, our study aimed to elucidate how putatively altered chromatin filters in aging cells contribute to their distinct responses to mechanical and growth factor signaling, manifesting as divergent gene expression patterns.

In this study, we embedded human dermal fibroblasts from different age groups into 3D collagen matrices and subjected them to mechanical tension and/or TGF-β signals. Our findings revealed that young cells, in line with previous findings, displayed a more vigorous reaction to TGF-β, as evidenced by the activation of a greater number of genes, predominantly enriched in pathways related to extracellular matrix (ECM) organization and TGF-β signaling. Importantly, we found that matrix tension enhanced TGF-β stimulation in young cells, a response that was not observed in older cells. We identified that Activator Protein 1 (AP-1) plays a pivotal role in the chromatin accessibility remodeling induced by TGF-β and aging, while other age-specific responses are induced through interactions with TFs exhibiting age-specific activity. Collectively, our findings provide compelling evidence that chromatin states, functioning as dynamic signaling filters, play a crucial role in regulating nuclear mechanotransduction and gene expression responses to combined mechanical and biochemical stimuli. This study underscores how age-related alterations in chromatin contribute to divergent cellular behaviors and identifies AP-1 and age-specific interacting TFs as promising targets for therapeutic interventions and rejuvenation strategies aimed at mitigating the functional declines associated with aging.

## Results

### Aging Differentiates Fibroblasts’ Response to Matrix Tension.

**Fig. 1.**
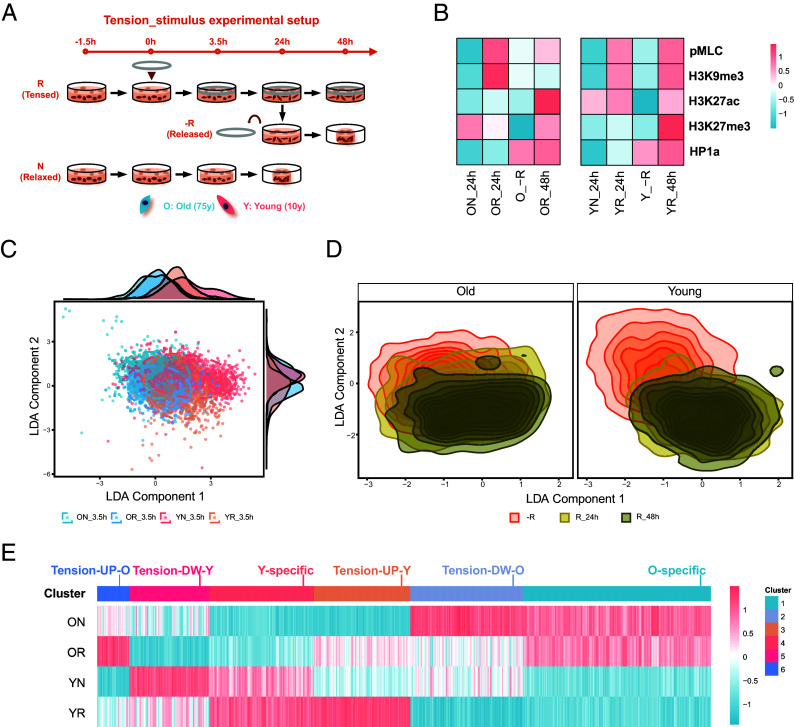
Aging differentiates fibroblasts’ response to matrix tension. (*A*) Tension-stimulus experimental setup. Human dermal fibroblasts from two age groups (young: 10 y, Y; old: 75 y, O) are seeded separately in collagen gel, followed by tension stimulus (addition of a mechanical ring). R: tensed matrix with ring; N: relaxed matrix without ring; −R: released matrix with ring removal at 24 h. (*B*) Heatmaps of mean normalized protein marker expression, scaled by row. Values are normalized by ON_24h for old and YN_24h for young cells. Unscaled values are in *SI Appendix*, Fig. S1. (*C*) Linear discriminant analysis (LDA) of nuclear features of fibroblasts in relaxed or tensed matrix for 3.5 h. Each dot represents one nucleus. (*D*) LDA plot with kernel density estimation showing the nuclear clustering density map of fibroblasts in tensed or released matrices for 24 h and 48 h. (E) Hierarchical clustering of gene expression of fibroblasts in tensed or relaxed matrices for 24 h. The values are the scaled log2(RPM+1) of all DE (differentially expressed, adj.*P* value <0.01, |log2FoldChange| > 1) genes from 6 pairwise comparisons, i.e., OR vs. ON, OR vs. YN, OR vs. YR, ON vs. YN, ON vs. YR, YR vs. YN. RPM: reads per million.

To investigate whether the cellular response to mechanical stimulus is age dependent, we seeded fibroblasts from two age groups, i.e., 10 y old (Y: Young) and 75 y old (O: Old), within 3D collagen gels. Mechanical tension was applied to the gels through a glass ring, which prevented the gel from shrinking, and the cellular responses were measured (Fig. 1*A* and *SI Appendix*, Fig. S1*A*). Without mechanical tension, the collagen gels gradually shrank (in the relaxed, without ring groups), while constraining the matrix with the glass ring (in the tensed, with ring groups) stopped the gels from shrinking (*SI Appendix*, Fig. S1*B*). Fibroblasts in the tensed conditions experienced higher matrix tension due to the fact that cells were experiencing higher counterforce from the collagen fibers that the fibroblasts intended to pull during spreading or migration. We compared the phenotypic changes of young and old fibroblasts under this mechanical tension for short-term (3.5 h) and long-term (24 h and 48 h) periods. The tensed matrix caused by the glass ring did not lead to apoptosis after 24 h, as evident by the low level of DRAQ7 staining (*SI Appendix*, Fig. S1*C*); meanwhile, it induced higher Ki67 expression (*SI Appendix*, Fig. S1*D*), indicating that cells in the tensed group are in a more proliferative state. pMLC was elevated in both age groups when cells sensed the tensed matrix for 3.5 h (*SI Appendix*, Fig. S1*E*), indicating higher cellular contraction than in the relaxed condition. Mechanical tension for 3.5 h also induced alterations in histone modifications, including the upregulation of H3K9me3, H3K27me3, and HP1a in both age groups, and reduction of H3K27ac in young cells (*SI Appendix*, Fig. S1 *F*–*I*), showing the potential for gene expression alterations.

To further investigate the cellular response to alterations of mechanical stimulus, we removed the glass ring from the 24 h tensed matrix (called the released group). Old cells retained contractility after being released from the tensed matrix, with pMLC expression similar to that of cells that had been in the tensed matrix for 48 h (Fig. 1*B* and *SI Appendix*, Fig. S1*J*). For the old cells, among the four histone modification markers we tested, H3K9me3, H3K27ac, and HP1a levels were maintained while H3K27me3 decreased after releasing the matrix tension. However, cell contraction and H3K9me3, H3K27ac, and HP1a levels decreased, while H3K27me3 levels were maintained in young cells once the ring was removed and matrix tension was released (Fig. 1*B* and *SI Appendix*, Fig. S1 *K*–*N*).

A LDA model based on chromatin texture and nuclear morphometric features (20) revealed that aging, in addition to matrix tension, impacted the cell states and cell response in each condition (Fig. 1*C*). These features showed a distinct shift in how the different aged cells responded when the ring was removed from the tensed matrices. All old cells, both those in the tensed matrix and those in the released matrix, clustered closely together, indicating that old cells did not experience an obvious change in their cell state once matrix tension was released. However, young cells in the released matrix clustered away from young cells in the tensed matrix, indicating that releasing matrix tension caused young cells to alter their cell states and change their behavior (Fig. 1*D*).

To further understand the state of these cells and how this might impact cell behavior, we performed unsupervised feature clustering (combining the nuclei from all of the cellular conditions) and found that the cells separated into four clusters based on nuclei size, shape, and heterochromatin content (HC) (*SI Appendix*, Fig. S2 *A*, *B*, and *D*). The first cluster (Large, HC low) involved nuclei with large projected area and low HC; the other three clusters involved nuclei with small projected area while differing in nuclear circularity (Small, Circular, HC low), elongation (Small, Elongated, HC low), and HC (Small, HC high). The fraction of these four clusters within each group (combination of matrix tension and age) varied by group (*SI Appendix*, Fig. S2*C*). Matrix tension did not change the nuclear aspect ratio, but old cells increased their nuclear elongation when matrix tension was released, and young cells decreased their nuclear circularity when matrix tension was released (*SI Appendix*, Fig. S2 *E*–*G*). The nuclear projected area was elevated in both old and young cells in the tensed matrix, while it decreased in old cells but remained constant in young cells when tension was released (*SI Appendix*, Fig. S2*H*). The different nuclear shapes between old and young cells suggest that aged cells respond differently to alterations in matrix tension. Thus, matrix tension variation induces differential histone modifications and nuclear morphological dynamics in young and old fibroblasts.

To determine what functional changes are induced by these differential cell states, we then checked the gene expression of fibroblasts in the relaxed and tensed matrices at the 24 h timepoint via bulk RNA-seq. Differentially expressed (DE) genes (adj.*P* value < 0.01, |log2FoldChange| > 1) gained from all pairwise comparisons (6 comparisons among four biological samples) could be hierarchically clustered into groups based on the gene expression alteration due to aging and/or mechanical stimulus (Fig. 1*E*). There were clusters of genes that did not respond significantly to matrix tension and were only highly expressed in either the young or old cells, indicated as Young-specific or Old-specific, respectively. The remaining gene clusters were affected by matrix tension. These genes could be further divided into age-specific subgroups that were elevated by matrix tension (Tension-UP-O and Tension-UP-Y) or suppressed by matrix tension (Tension-DW-O and Tension-DW-Y). The Young-specific genes were enriched in pathways related to developmental programmes and small GTPase activity, while Old-specific genes were enriched in limb morphology and BMP pathways (*SI Appendix*, Fig. S2*I*). Tension-UP-Y genes were enriched in proliferation pathways, while Tension-UP-O genes were enriched in cell migration and protein synthesis. Tension-DW-Y genes were enriched in cell morphogenesis, and Tension-DW-O genes were enriched in ECM organization and immune response. These findings indicate that young fibroblasts adapted to the tensed matrix more likely via increasing proliferation, while old fibroblasts adapted by elevating cell migration. Overall, aging-induced variations in the gene profile of fibroblasts led to divergent cellular responses to the same mechanical stimulus.

### Matrix Tension Promotes Young Fibroblasts’ Response to TGF-β.

**Fig. 2.**
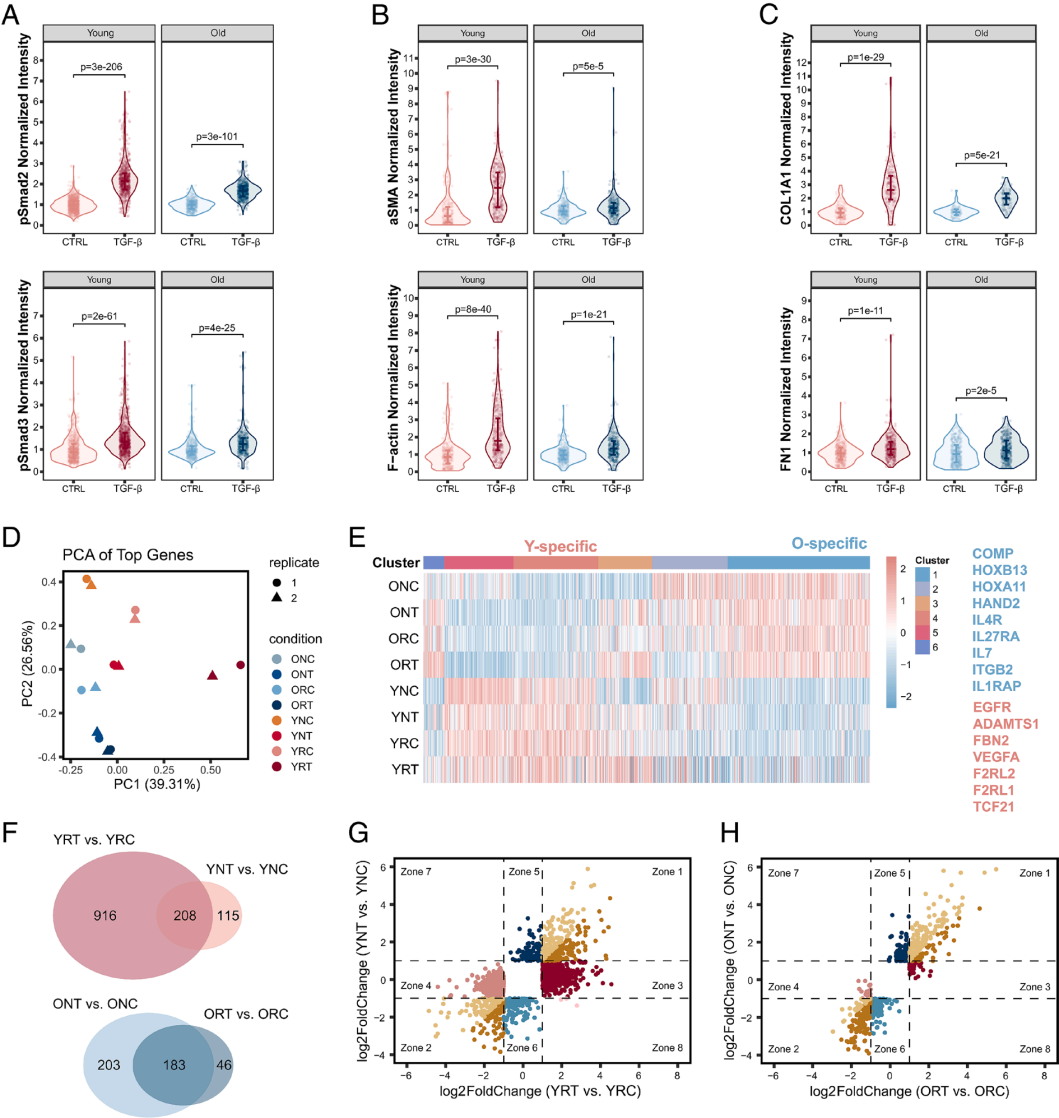
Matrix tension promotes young fibroblasts’ responses to TGF-β. (*A*–*C*) Violin plots of normalized fibroblast protein markers in tensed matrix with/without TGF-β. (*A*) phospho-Smad2 (pSmad2, n = 3) and phospho-Smad3 (pSmad3, n = 3), (*B*) alpha-smooth muscle actin (aSMA, n = 6) and F-actin (n = 5), (*C*) COL1A1(n = 5) and FN1(n = 3). Fibroblasts in tensed matrix without TGF-β (CTRL) and with TGF-β (TGF-β). Expression values are normalized to CTRL means. All data points are shown; quartiles are indicated. *P*-values are from two-sided Wilcoxon tests. (*D*) First (x-axis, PC1) and second (y-axis, PC2) components of a PCA of RNA-seq data from fibroblasts in Tension/TGF-β-stimulus conditions. NC: with neither tension nor TGF-β stimulus; NT: without tension, with TGF-β stimulus; RC: with tension, without TGF-β stimulus; RT: with tension and TGF-β stimulus. Eight conditions with two biological replicates. (*E*) Gene expression heatmap of overlapped DE genes in Tension-stimulus conditions (from Fig. 1*E*) and Tension/TGF-β-stimulus conditions. Clusters are consistent with those from Fig. 1*E*; gene expression values are the scaled log_2_(RPM+1) in Tension/TGF-β-stimulus conditions. Listed genes are O-specific (blue) and Y-specific genes (pink). (*F*) Venn diagrams showing overlap of TGF-β-induced upregulated genes (adj.*P* value < 0.01, log_2_FoldChange > 1) between tensed (R) and relaxed (N) conditions. YRT vs. YRC: upregulated genes in YRT vs. YRC; YNT vs. YNC: upregulated genes in YNT vs. YNC; ORT vs. ORC: upregulated genes in ORT vs. ORC; ONT vs. ONC: upregulated genes in ONT vs. ONC. (*G* and *H*) Scatter plots of TGF-β-induced DE genes in (*G*) young and (*H*) old cells with tension (x-axis) and without tension (y-axis). Colors are used to separate genes located in different coordinate zones (Zone).

As the cellular age resulted in differential responses to mechanical stimuli, we next introduced TGF-β to investigate if aging would also influence the fibroblast response to the same biochemical external stimulus, both with and without matrix tension. We performed a parametric study to examine the effects of age (Y: young or O: old), tension (R: tensed or N: relaxed), and TGF-β treatment (T: TGF-β or C: control). In the canonical TGF-β pathway, Smad2 and Smad3 are phosphorylated and translocated into the nucleus to further induce fibroblast differentiation into myofibroblasts and modulate the ECM (23). In both young and old fibroblasts, TGF-β increased nuclear phospho-Smad2 (pSmad2) and phospho-Smad3 (pSmad3) levels, showing that fibroblasts of both age conditions were stimulated (Fig. 2*A* and *SI Appendix*, Fig. S3*A*). To verify the activation levels of the TGF-β-treated fibroblasts, we measured myofibroblast markers (aSMA, F-actin) and ECM markers (COL1A1, FN1). Both age groups showed higher levels of these proteins with TGF-β treatment. In addition, young cells exhibited higher activation in response to TGF-β compared to old cells, with a greater elevation in the expression of aSMA, F-actin (Fig. 2*B* and *SI Appendix*, Fig. S3*B*), and COL1A1 (Fig. 2*C* and *SI Appendix*, Fig. S3*C*). In addition, single-cell correlation analysis in young fibroblasts shows high Pearson correlations between COL1A1 and α-SMA intensity, and between F-actin and COL1A1 intensity (*SI Appendix*, Fig. S12). This coincident expression suggests that the synergistic response in young cells is driven by tightly coupled mechanical and biochemical activation at the single cell level. However, the fibroblasts from the 96-y-old group showed no increase in aSMA, while middle-aged (39 y old) cells displayed moderate elevation of aSMA after TGF-β treatment, further supporting this finding (*SI Appendix*, Fig. S3*D*). We then checked the gene expression among age and treatment (TGF-β and matrix tension) conditions using bulk RNA-seq to understand how aging influences cellular responses. A principal component analysis (PCA) of the top 2,000 most variable genes revealed that gene expression is separable by age, TGF-β, and matrix tension, with substantial differences between dual-stimulated young cells (YRT) and the other treatment groups (Fig. 2*D*). We checked how the DE genes from Tension-stimulus conditions varied in the Tension/TGF-β-stimuli conditions (Fig. 2*E*), and interestingly found that age specificity was maintained (e.g., COMP, HOXB13, IL7, IL1RAP expressed higher in old, and EGFR, ADAMTS1, FBN2, TCF21 expressed higher in young cells, regardless of the Tension/TGF-β treatments). Among the 1,124 upregulated genes in the YRT vs. YRC comparison group, 208 genes overlapped with the 323 upregulated genes in the YNT vs. YNC comparison; among the 229 upregulated genes in the ORT vs. ORC comparison, 183 genes overlapped with the 386 upregulated genes in the ONT vs. ONC comparison, suggesting that mechanical tension has less of an impact on the signaling response of old cells than of young cells (Fig. 2*F*); likewise, a greater effect of tension on young cells compared to old cells was observed in the downregulated genes as well (*SI Appendix*, Fig. S3*E*).

Scatter plots further distinguished how matrix tension influenced young and old fibroblasts’ responses to TGF-β (Fig. 2 *G* and *H*). TGF-β changed a larger number of genes in young cells (Fig. 2*G*, 600 DE genes in YNT vs. YNC; 2,128 DE genes in YRT vs. YRC) than old ones (Fig. 2*H*, 738 DE genes in ONT vs. ONC; 391 DE genes in ORT vs. ORC). In both young and old cells, there was a portion of genes regulated by TGF-β in the same direction, no matter which matrix conditions the cells resided in (genes in Zones 1 and 2). Other genes responded to TGF-β only in the tensed matrix (genes in Zones 3 and 4), and some responded only in the relaxed matrix (genes in Zones 5 and 6). In young cells, significantly more genes were activated only when the cells were in the tensed matrix, which means that TGF-β activated more genes under tensed conditions in young fibroblasts than in old fibroblasts.

Genes upregulated by matrix tension without TGF-β in young cells were enriched in proliferation-related pathways (*SI Appendix*, Fig. S4*B*), aligning with the results from Tension-UP-Y gene cluster in Fig. 1*E* and *SI Appendix*, Fig. S4. The introduction of TGF-β to these matrices of young cells showed that TGF-β induced ECM organization and TGF-β response pathways in both the relaxed and tensed matrices (*SI Appendix*, Fig. S4 *A* and *D*), with the tensed matrix further promoting responses in these pathways (*SI Appendix*, Fig. S4*C*). Among the genes that are highly upregulated by TGF-β in young cells with tension were many ECM genes, including the metalloproteinase such as MMP2, MMP13, MMP14, ADAM19, ADAM11, ADAMTS7, ADAMTS16, ADAMTS2, ADAMTS4, and collagens such as COL4A1, COL4A2, COL9A2, COL1A2, COL16A1, COL5A1, COL7A1, COL1A1 (*SI Appendix*, Fig. S3*F*). TGF-β treatment in old cells primarily induced metabolic and developmental pathways in the relaxed matrix (*SI Appendix*, Fig. S4*E*), while the tensed matrix promoted more collagen fiber organization processes responding to TGF-β (*SI Appendix*, Fig. S4*H*) and stress response pathways (*SI Appendix*, Fig. S4 *F* and *G*). Thus, the tensed matrix enhanced young fibroblasts’ responses to TGF-β compared to old fibroblasts by activating more genes and promoting ECM organization and TGF-β response processes.

We examined variations in the expression of epigenetic modifiers, identifying aging-specific ones, such as NPAS2, MBNL3, BARD1, and CELF2, which were more upregulated in young cells, and PARP3 and SMYD3, which were more upregulated in old cells. Additionally, we found tension-responding modifiers that were upregulated in both cell types within the tensed matrices, including DNMT1, PRDM16, HR, RAD51, HELLS, and ATAD2 (*SI Appendix*, Fig. S3*G*). For instance, RAD51 and HELLS were upregulated in both cell types within the tensed matrix, which may be attributed to the role of RAD51 in homologous recombination and the protection of stressed DNA (24), as well as the role of HELLS in DNA repair and maintaining genome stability (25). BARD1 exhibits higher expression in young cells compared to old cells, potentially aiding DNA repair and calcium signaling more effectively in young cells (26, 27). On the contrary, SMYD3, which was higher in old cells, could promote senescence-associated phenotypes (28). Similar phenotypes were observed in adhesome genes (*SI Appendix*, Fig. S3*H*). The young-specific gene DLC1 is noted as an attenuator for Rho-GTP activation, which aligns with the enrichment of Rho-GTP pathway in young cells (29, 30). Additionally, SDC4 is identified as a gene responsive to force, aligning with findings that SDC4 adjusts cellular mechanics in reaction to local tension by activating the kindlin-integrin-RhoA pathway (31). Additionally, an age-specific response was evident in the expression of target genes for Smad2, Smad3, and Smad4, which are key TFs of TGF-β signaling; moreover, tension itself induced variation in the expression of these target genes (*SI Appendix*, Fig. S3*I*). A similar observation was found for TFs and mechanical stimulus-related genes (*SI Appendix*, Fig. S3 *J* and *K*). Some mechanical stimulus-related genes were involved in TGF-β signaling (i.e., MMP2, JUN, MMP14, COL1A1, TGFB1, JUND, FOS, FOSL1, SOX9). The age specificity of epigenetic modifiers, adhesomes, Smad 2/3/4-target genes, and TFs, as well as the mechanical stimulus-related genes, may contribute to the tension-promoted response of fibroblasts to TGF-β.

### Age-Specific Gene Sets Get Activated by TGF-β in Young and Old Fibroblasts.

**Fig. 3.**
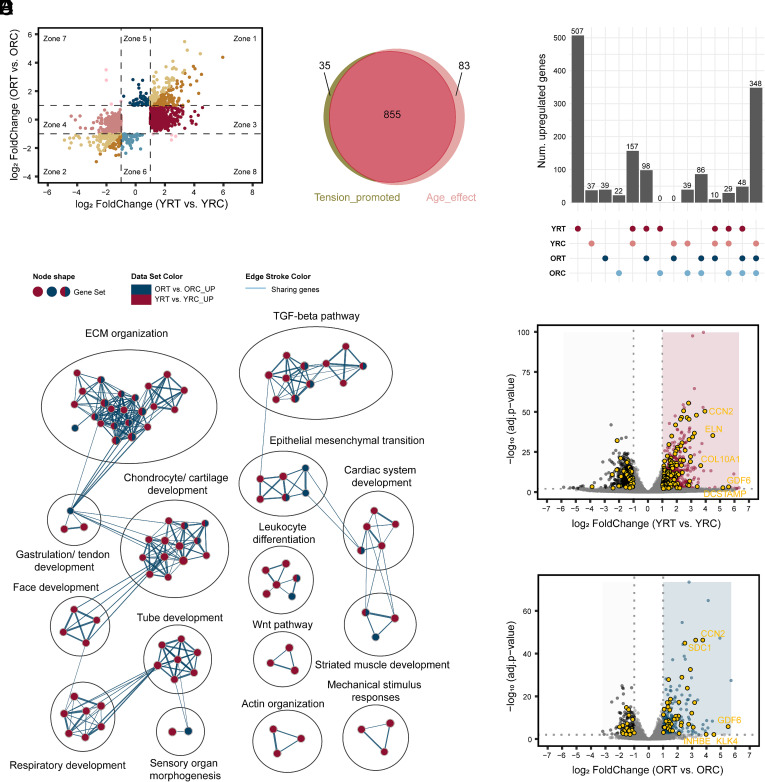
Age-specific gene sets get activated by TGF-β in young and old fibroblasts. (*A*) Scatter plots of TGF-β-induced DE genes in young (x-axis) and old (y-axis) cells with tension. Colors are used to separate genes located in different coordinate zones (Zone). (*B*) Venn diagram showing the overlap of Tension_promoted genes (genes in Zone 3 of Fig. 2*G*) and Age_effect genes (genes in Zone 3 of Fig. 3*A*). (*C*) Upset plot showing overlaps among upregulated genes in YRT, YRC, ORT, and ORC conditions. Upregulated genes in each condition are DE genes with log_2_FoldChange > 1 relative to the other three conditions. (*D*) Enrichment pathway network showing pathways from upregulated genes in YRT vs. YRC and ORT vs. ORC. Each dot represents an enriched pathway; colors: pathway involving upregulated genes in YRT vs. YRC (red), ORT vs. ORC (blue), or both comparisons (red and blue). Edge: gene overlap between pathways, with thickness indicating the Jaccard Overlap. Selected pathway clusters are shown; details in Datasets S1 and S2. (*E* and *F*) Volcano plots of DE genes in (*E*) YRT vs. YRC and (*F*) ORT vs. ORC with ECM organization and TGF-β pathways genes labeled in yellow. Red dots: upregulated genes in YRT vs. YRC; blue dots: upregulated genes in ORT vs. ORC; black dots: downregulated genes; yellow dots: pathway genes; gray dots: non-DE genes.

Given that fibroblasts commonly experience the interplay of biochemical and mechanical stimuli, we further focused on how TGF-β stimulus affects cells in the tensed matrix. 192 genes (Zone 1) were upregulated, and 120 genes (Zone 2) were downregulated in both young (YRT) and old (ORT) fibroblasts after TGF-β treatment (Fig. 3*A*). In the YRT condition, 938 genes (Zone 3) were upregulated, while 884 genes (Zone 4) were downregulated, with no significant fold change observed in ORT. In comparison, 56 genes (Zone 5) were upregulated and 57 genes (Zone 6) were downregulated in ORT, showing no significant changes in YRT (Fig. 3*A*). Among the 938 upregulated genes in young fibroblasts, we found that 855 were responsive to TGF-β only in the tensed matrix, not in the relaxed matrix (Fig. 3*B*, comparing Fig. 2*G*, Zone 3 and Fig. 3*A*, Zone 3). This highlights that matrix tension enhances the response of young cells to TGF-β.

Comparing the four tensed matrix conditions (i.e., YRT, YRC, ORT, and ORC), we further identified 507 genes with the highest expression and 348 genes with the lowest expression in YRT (Fig. 3*C*). In contrast, fewer than 40 genes were highly expressed in each of the other three conditions, with 348 genes shared among them. Thus, TGF-β regulated many genes that were uniquely responsive in young fibroblasts in the tensed matrix. To further investigate the expression changes of genes upregulated by TGF-β, we identified six clusters that differentiate the aging response to TGF-β through hierarchical clustering (*SI Appendix*, Fig. S5*A*). Cluster 1 and 4 genes, including COL1A1, LIMK2, TGFB1, FOXJ2, LTBP3, and SKI, were activated by TGF-β only in young cells; Cluster 2 and 3 genes, including JUNB, SOX9, CCN2, and ELN, responded to TGF-β in both young and old cells; and Cluster 5 and 6 genes, including LIMS1, ID4, ASPN, and PITX2, were elevated by TGF-β only in old cells (*SI Appendix*, Fig. S5 *B* and *C*).

To understand the functions of these altered genes, we performed a pathway enrichment network analysis (32) for upregulated and downregulated genes from YRT vs. YRC and ORT vs. ORC (Fig. 3*D* and *SI Appendix*, Fig. S5 *D* and *E*). Upregulated genes in both YRT and ORT were most enriched in ECM regulation and TGF-β response pathways (Fig. 3*D* and *SI Appendix*, Fig. S5*D* and Datasets S1 and S2). In YRT, upregulated genes were also enriched in developmental pathways, actin organization pathways, and mechanical stimulus response pathways, which were rarely found in ORT. Downregulated genes were not as functionally enriched as upregulated genes (*SI Appendix*, Fig. S5*E* and Dataset S3 and S4). Considering TGF-β is an important factor for ECM modulation and fibroblasts are key regulators of the ECM in the skin, these results indicate that the upregulated genes in young fibroblasts were important for fibroblast function and required for maintenance of tissue homeostasis. Therefore, the genes enriched in ECM regulation and TGF-β response pathways were identified as key genes associated with the aging effect. Consistently, more genes in YRT were enriched in these two categories than in ORT, with more upregulated genes than downregulated genes in both YRT and ORT (Fig. 3 *E* and *F*). Though some pathway-related genes were responding in the same direction in both young and old cells, more genes only responded in young cells (*SI Appendix*, Fig. S5*F*). For instance, ACTA2, which encodes aSMA, was upregulated in both young and old cells, while some ECM and TGF-β response genes (e.g., COL1A1, FN1, MMP13, TGFB1, LTBP2) were only upregulated in young cells. Therefore, the stimulation of fibroblasts by TGF-β demonstrated a significant age bias.

### TGF-β and Age Affect Chromatin Accessibility.

**Fig. 4.**
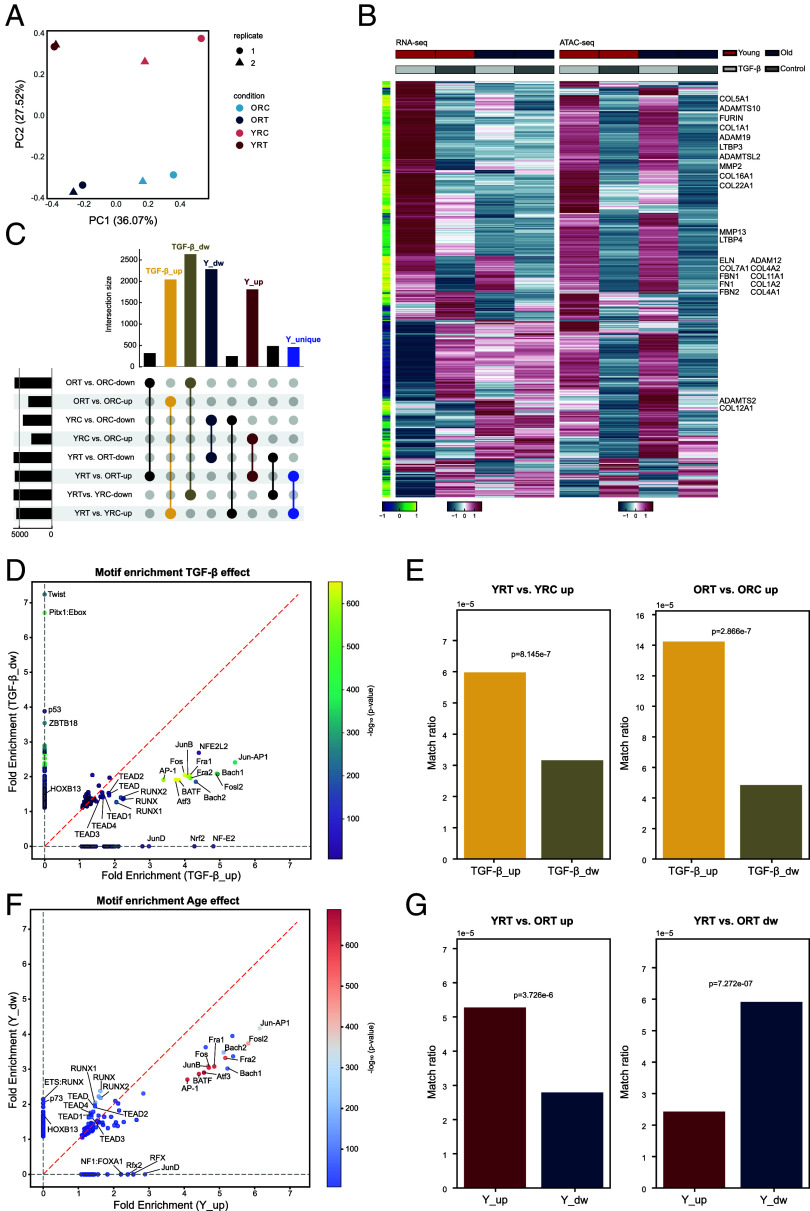
TGF-β and age affect chromatin landscape. (*A*) First (x-axis, PC1) and second (y-axis, PC2) principal components for four conditions’ ATAC-seq data, i.e., YRT, YRC, ORT, and ORC, in two biological replicates. (*B*) Concordance in gene expression and chromatin accessibility patterns. Expression levels (*Left*, scaled log_2_(RPM+1)) and ATAC counts at the peak 20 kb upstream or downstream of the gene body (*Right*, z score of normalized counts) for DE genes (rows) with the highest correlation of accessibility signal with gene expression. The green column on the left shows the Pearson correlation coefficient for gene expression and ATAC-seq data. (*C*) Upset plot showing the differential accessible regions (DACRs) overlaps among all pairwise comparisons. Selected categories showing the overlap between two pairwise comparisons are displayed. TGF-β effect: overlapping DACRs between YRT vs. YRC and ORT vs. ORC, including TGF-β_up effect and TGF-β_dw effect (TGF-β_up effect indicates overlapping DACRs between YRT vs. YRC-up DACRs and ORT vs. ORC-up DACRs. TGF-β_dw effect indicates overlapping DACRs between YRT vs. YRC-down DACRs and ORT vs. ORC-down DACRs); Age effect: overlapping DACRs between YRT vs. ORT and YRC vs. ORC, including Y_up effect and Y_dw effect (Y_up effect indicates overlapping DACRs between YRT vs. YRC-up DACRs and ORT vs. ORC-up DACRs. Y_dw effect indicates overlapping DACRs between YRT vs. YRC-down DACRs and ORT vs. ORC-down DACRs). (*D*) Scatter plot of enriched motifs on TGF-β effect DACRs (detailed comparison groups shown in *C*). Each dot indicates one motif with dot color representing −log_10_(*P*-value). The red dashed line indicates the diagonal. (*E*) Bar plots showing the upregulated gene and motif match ratio. Motifs enriched in TGF-β_up DACRs show a greater match ratio with TGF-β-induced upregulated genes, i.e., upregulated genes in YRT vs. YRC (*Left* panel) and ORT vs. ORC (*Right* panel), than motifs in TGF-β_dw DACRs. *P*-values are calculated by the Proportion Z-Test. (*F*) Scatter plot of enriched motifs on Age effect DACRs (detailed comparison groups shown in *C*). Each dot indicates one motif with dot color representing −log_10_(*P*-value). The red dashed line indicates the diagonal. (*G*) Bar plots showing the upregulated gene and motif match ratio. Match ratio is the motif-binding upregulated genes divided by total upregulated genes, normalized by total motif-binding genes. Motifs enriched in Y_up DACRs show a greater match ratio with upregulated genes in young cells, i.e., upregulated genes in YRT vs. ORT (*Left* panel), than motifs in Y_dw DACRs; motifs in Y_dw DACRs show a greater match ratio with downregulated genes in old cells YRT vs. ORT (*Right* panel) than motifs in Y_up DACRs. *P*-values are calculated by the Proportion Z-Test.

To understand the chromatin states of young and old fibroblasts, we performed ATAC-seq on fibroblasts in the tensed matrices, both with and without TGF-β treatment. We focused our chromatin accessibility profiling on fibroblasts in tensed matrices, as mechanical tension is a prerequisite for TGF-β-mediated myofibroblast differentiation and best recapitulates the physiological mechanical environment. PCA of the ATAC-seq results revealed substantial differences in the chromatin landscapes of young and old fibroblasts, as well as between those treated with TGF-β and those without (Fig. 4*A*). The first principal component (PC1) consistently reflected changes induced by TGF-β treatment in both young and old cells, while the second principal component (PC2) distinguished between young and old cells, regardless of TGF-β treatment. Most ATAC-seq peaks were within 1 kb width and were annotated to only one gene (*SI Appendix*, Fig. S6 *A* and *B*). More than 97% of peaks were within 200 kb distance to a TSS (transcription start site): 31.77% of peaks were within 2 kb distance, 36.4% within 2 to 20 kb, 29.59% within 20 to 200 kb (*SI Appendix*, Fig. S6 *C* and *D*). To relate gene expression and chromatin accessibility, we correlated the RNA-seq expression data and ATAC-seq peak data (Fig. 4*B*). Some genes were highly positively correlated in both young and old cells, such as FN1, COL1A2, and COL4A2. Additionally, some genes with open peaks in both young and old cells exhibit gene upregulation only in young or old cells, such as COL1A1, which is upregulated in young cells (*SI Appendix*, Fig. S6*E*). This suggests the possibility of diminished activity or recruitment efficacy of critical TFs at these open loci, underscoring the need to dissect the specific differences in TF activity between young and old fibroblasts.

To comprehensively analyze the alterations in chromatin accessibility, we concentrated on the differentially accessible chromatin regions (DACRs). DACRs were predominantly found in intronic and intergenic regions, situated more than 25,000 bp away from the nearest TSS (*SI Appendix*, Fig. S6 *F* and *G*). This emphasizes the importance of distal regulatory elements in distinguishing and potentially regulating age-related TGF-β responses and senescence. The vast majority of DE genes (3,131 out of 3,476) were associated with open peaks (*SI Appendix*, Fig. S6*H*), and nearly half (1,648 out of 3,476) of the DE genes were associated with DACRs (*SI Appendix*, Fig. S6*I*). Within the DACRs identified from a total of eight pairwise comparisons, several categories encompass more than 1,500 DACRs (*SI Appendix*, Fig. S6*J*). Notably, these categories included DACRs induced by TGF-β treatment across both age groups (TGF-β-induced more accessible regions: TGF-β_up, and TGF-β-induced less accessible regions: TGF-β_dw), as well as those influenced by aging irrespective of TGF-β treatment (more accessible regions in young cells: Y_up, and more accessible regions in old cells: Y_dw, Fig. 4*C* and *SI Appendix*, Fig. S6*J*).

Strikingly, AP-1 related TFs (i.e., Jun-AP1, Fosl2, Bach1, Bach2, Fra1, Fra2, Fos, JunB, BATF, ATF3, and AP-1) appear as the most significantly enriched motifs in both more and less accessible chromatin regions induced by TGF-β. TGF-β_up regions were particularly enriched with TF binding sites that mediate cellular stress responses, including Nrf2 and Mafk (33, 34). In contrast, TGF-β_dw regions were characterized by an enrichment of TF binding sites involved in epithelial–mesenchymal transition (EMT) and tumor metastasis, such as Twist, Pitx1, and p53 (35–37) (Fig. 4*D*). Based on the established roles of these factors in tumor suppression and cell identity, the reduced accessibility at these motifs likely represents the silencing of genes that normally restrict cell proliferation and plasticity. Motifs enriched in TGF-β-induced more accessible regions regulated a significantly higher ratio of upregulated genes in both young and old fibroblasts compared to motifs enriched in TGF-β-induced less accessible regions (Fig. 4*E*). The downregulated genes in young and old cells were more strongly associated with motifs enriched in less accessible regions than with motifs in more open regions (*SI Appendix*, Fig. S7*A*). The AP-1 motifs enriched in more open peaks also exhibited greater regulation with the upregulated genes compared to the AP-1 motifs enriched in less open peaks (*SI Appendix*, Fig. S7*B*). The same phenomenon was observed for RUNX and TEAD TFs (*SI Appendix*, Fig. S7*C*).

Interestingly, AP-1 related TFs were the most enriched motifs found in both more and less accessible chromatin regions differentiated by age as well (Fig. 4*F*). The ATAC-seq peaks that were more open in young cells are enriched with sites of Forkhead domain TFs, such as FOXA3, which can regulate metabolic homeostasis and oxidative stress (38, 39). The peaks that are uniquely more open in old cells are enriched with sites of Homeobox domain TFs. The motifs present in the more open areas of young cells showed a stronger association with young upregulated genes than those found in the less open regions of young cells. In contrast, motifs from the less open regions of young cells were more closely linked to upregulated genes in old cells compared to motifs from the more open regions of young cells (Fig. 4*G*). This trend is also valid for peaks containing motifs of the AP-1 family or the RUNX/TEAD families, where motifs in the Y_up regions primarily regulated more young upregulated genes, while motifs in the Y_dw regions regulated more old upregulated genes (*SI Appendix*, Fig. S7 *D* and *E*). This increased regulation of upregulated genes associated with motifs in more accessible regions was observed in both TGF-β-induced and age-induced regions. Given that the AP-1 TFs were enriched in both TGF-β- and age-altered chromatin regions, these results highlight the convergence of aging and the TGF-β response via the AP-1 TFs.

To further investigate different TF motifs enriched in age-induced DACRs, in addition to AP-1, we clustered motifs based on motif enrichment analysis using Y_up and Y_dw DACRs (Fig. 4*F*). Four motif groups were identified (Dataset S5), i.e., the AP-1 group (highly enriched in both Y_up and Y_dw DACRs), the RUNX/TEAD group (moderately enriched in both Y_up and Y_dw DACRs), the Y-specific motifs (exclusively enriched in Y_up DACRs, e.g., JUND), and the O-specific motifs (solely enriched in Y_dw DACRs, e.g., HOXB13). We examined the proximity of upregulated genes in young cells related to motif binding sites in Y_up DACRs and downregulated genes associated with motifs in Y_dw DACRs. No significant proximity differences appeared among motif groups in each age condition (*SI Appendix*, Fig. S7*F*). Interestingly, nearly all Y_up DACRs possessing AP-1 and RUNX/TEAD motif binding sites associated with upregulated DE genes identified in YRT also contained Y-specific motif binding sites (*SI Appendix*, Fig. S7*G*). However, there were additional Y_up DACRs with upregulated DE genes that have exclusively Y-specific motif binding sites. Similarly, there were additional Y_dw DACRs with downregulated genes that have exclusively O-specific motif binding sites. This observation suggests potential collaborations between TFs exhibiting age-specific activity and AP-1, as well as RUNX/TEAD TFs, which are responsible for the transcriptional response to TGF-β during aging.

Herein, we identified the relative enrichment of specific motifs based on TGF-β- and age-dependent DACRs, with a particular emphasis on the dominant motifs belonging to the AP-1 family, as well as those that are specific to different age groups.

### AP-1 Motifs Dominate Chromatin’s Differential Accessibility for Gene Regulation.

**Fig. 5.**
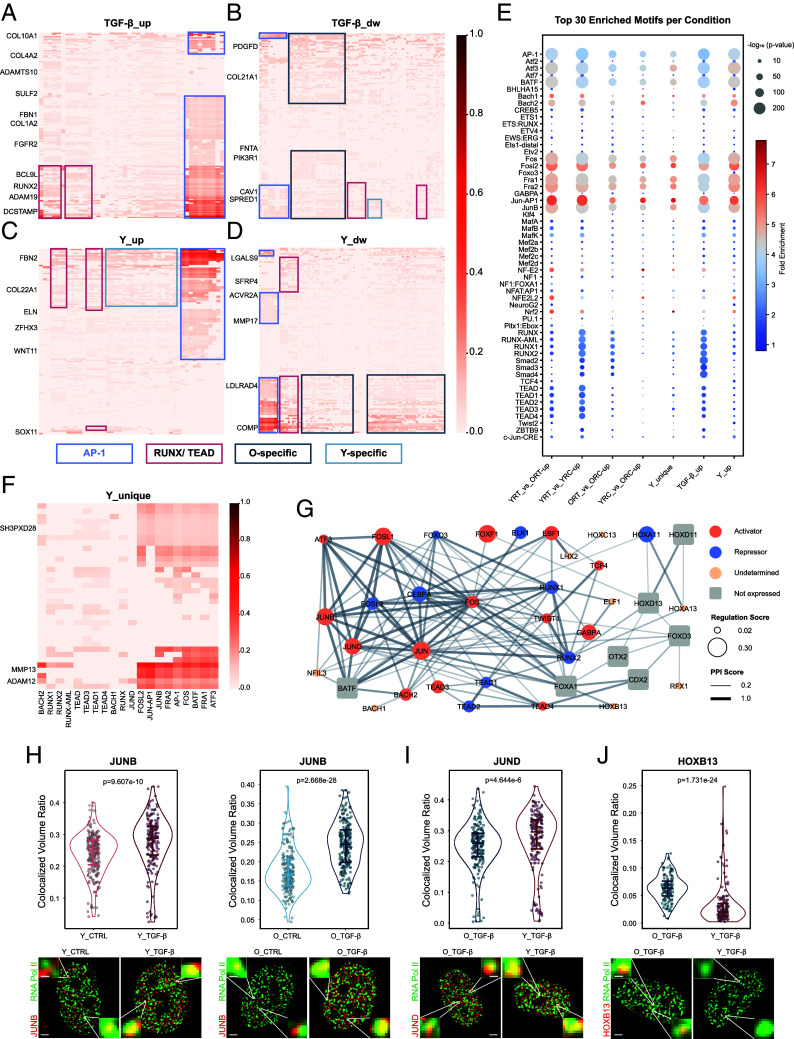
AP-1 motifs dominate chromatin’s differential accessibility for gene regulation. (*A*–*D*) Motif-gene regulation weight score heatmaps of (*A*) TGF-β-up, (*B*) TGF-β-dw, (*C*) Y-up, and (*D*) Y-dw effects (detailed comparison groups shown in Fig. 4*C*). Row: overlapping DE genes in two pairwise comparisons; row scores: scaled log2FoldChange mean values of two pairwise comparisons; column: motifs enriched in the category of DACRs; column scores: scaled Fold Enrichment of motif. Selected genes that are involved in ECM organization and TGF-β response pathways are labeled (complete heatmaps in *SI Appendix*, Fig. S8). Colored boxes label motif-gene modules for selected motifs (AP-1 motifs, RUNX/TEAD motifs, O-specific motifs; Y-specific motifs). (*E*) Enriched motifs in more accessible DACRs. Top 30 motifs in each category of DACRs are shown with dot size indicating −log_10_(*P*-value) and dot color indicating Fold Enrichment. (*F*) Motif-gene regulation weight score heatmap of Y-unique effect. Selected genes that are involved in ECM organization and TGF-β response pathways are labeled (complete heatmap in *SI Appendix*, Fig. S8). (*G*) Motif interaction network. Motif activations were calculated via. Dot: diffTF calculated motifs (orange: activator; blue: repressor; yellow: undetermined); Dot size: absolute median Pearson correlation values from diffTF results. Edge weight: protein–protein interaction scores (PPI scores) from STRING. Round square: motifs not expressed according to RNAseq. (*H*–*J*) Violin plots and representative images of colocalization of (*H*) JUNB, (*I*) JUND, and (*J*) HOXB13 with RNA polymerase II. Volume ratio: overlapped volume of TF and RNA polymerase II normalized by nuclear volume. *P*-values from two-sided Wilcoxon tests, n = 3. Representative images from Nikon DeepSIM. (Scale bar, 2 µm.) *Insets* zoom small white box regions. (Scale bar, 0.2 µm.)

To understand the importance and functions of enriched motifs, we examined the gene-motif weight scores calculated based on both gene expression and motif enrichment. We selected four motif groups (AP-1, RUNX/TEAD, Y_specific, and O_specific, see Dataset S5), all of which displayed individual gene-motif modules wherein the motif-gene pairs had higher weight scores compared to the background (*SI Appendix*, Fig. S8 *A*–*D* and Fig. 5 *A*–*D*). The AP-1 group motifs were affected by both TGF-β and age, and had high weight scores with genes, including ECM and TGF-β pathway related genes, in TGF-β_up, Y_up, and Y_dw. In contrast, the RUNX/TEAD group motifs were also affected by both TGF-β and age but exhibited lower weight scores with genes than AP-1 motifs. The Y-specific motifs module was found in Y_up and TGF-β_dw conditions, though the weight scores were relatively low. Furthermore, the O-specific motifs module was identified in Y_dw and TGF-β_dw conditions with low weight scores. Here, the AP-1 motifs demonstrated the highest weight scores with age-specific genes and TGF-β-upregulated genes, indicating that AP-1 motifs interact with distinct sets of genes across different age scenarios and were among the main factors responding to TGF-β.

Among the DACR categories identified from the eight pairwise comparisons, a significant proportion of DACRs manifested solely in one specific comparison, indicating condition specificity (*SI Appendix*, Fig. S6*J*). The enriched motifs for more accessible regions were mainly grouped in AP-1 families, with some TGF-β-effect motifs (e.g., Smad2, Smad3, Smad4, and ZBTB9) and age-effect motifs (e.g., TCF4, FOXO3, NF1:FOXA1) (Fig. 5*E*). Notably, one category (defined as Y_unique), comprised of more than 400 DACRs, pertained to the TGF-β-induced open peaks observed in young cells, which were comparatively more accessible than those in TGF-β-induced old cells (Fig. 4*C*). AP-1 group motifs enriched in these DACRs also showed relatively high weight scores to Y_unique DE genes (*SI Appendix*, Fig. S8*E* and Fig. 5*F*). Our findings reveal that AP-1 motifs were consistently found to be highly enriched and with high weight scores to DE genes across all categories of TGF-β- or Young-induced more accessible DACRs (*SI Appendix*, Figs. S8 and S9). Similarly, AP-1 motifs were highly enriched in Old-induced more accessible DACRs (*SI Appendix*, Fig. S10*A*). AP-1 motifs were enriched in TGF-β-induced less accessible DACRs as well, though Twist and Pitx1:Ebox were highly enriched.

To investigate the potential of TF coregulation events, we found that RUNX/TEAD motifs, Y-specific motifs, and O-specific motifs displayed a high co-occurrence among groups. AP-1 motifs were found to co-occupy motif sites of other groups more in TGF-β- and Young-induced more accessible DACRs than in less accessible DACRs (*SI Appendix*, Fig. S10 *B* and *C*). We also examined the regulatory relationship among the four motif groups by investigating the TFs’ activities (40). In the YRT condition, AP-1 motifs showed increased activity compared to the ORT condition (*SI Appendix*, Fig. S10 *D* and *E*). We also found that age-specific motifs exhibit higher activity in certain ages (e.g., JUND in YRT and HOXB13 in ORT). The regulatory network revealed that AP-1 motifs dominate and closely interact with motifs from other groups (Fig. 5*G*).

To experimentally validate the TFs’ activities, we costained JUNB (representative of AP-1 motifs), JUND (representative of Y-specific motifs), and HOXB13 (representative of O-specific motifs) with Ser 5 phosphorylated RNA polymerase II (RNA poly II s5) (Fig. 5 *H*–*J*). We found that JUNB colocalized more with RNA polymerase II s5 after TGF-β treatment in both young and old cells, while JUND displayed higher colocalization with RNA polymerase II s5 in young and HOXB13 showed higher colocalization in old cells. Altogether, we assessed the regulatory relationship between the binding motifs in accessible chromatin regions and the DE genes.

**Fig. 6.**
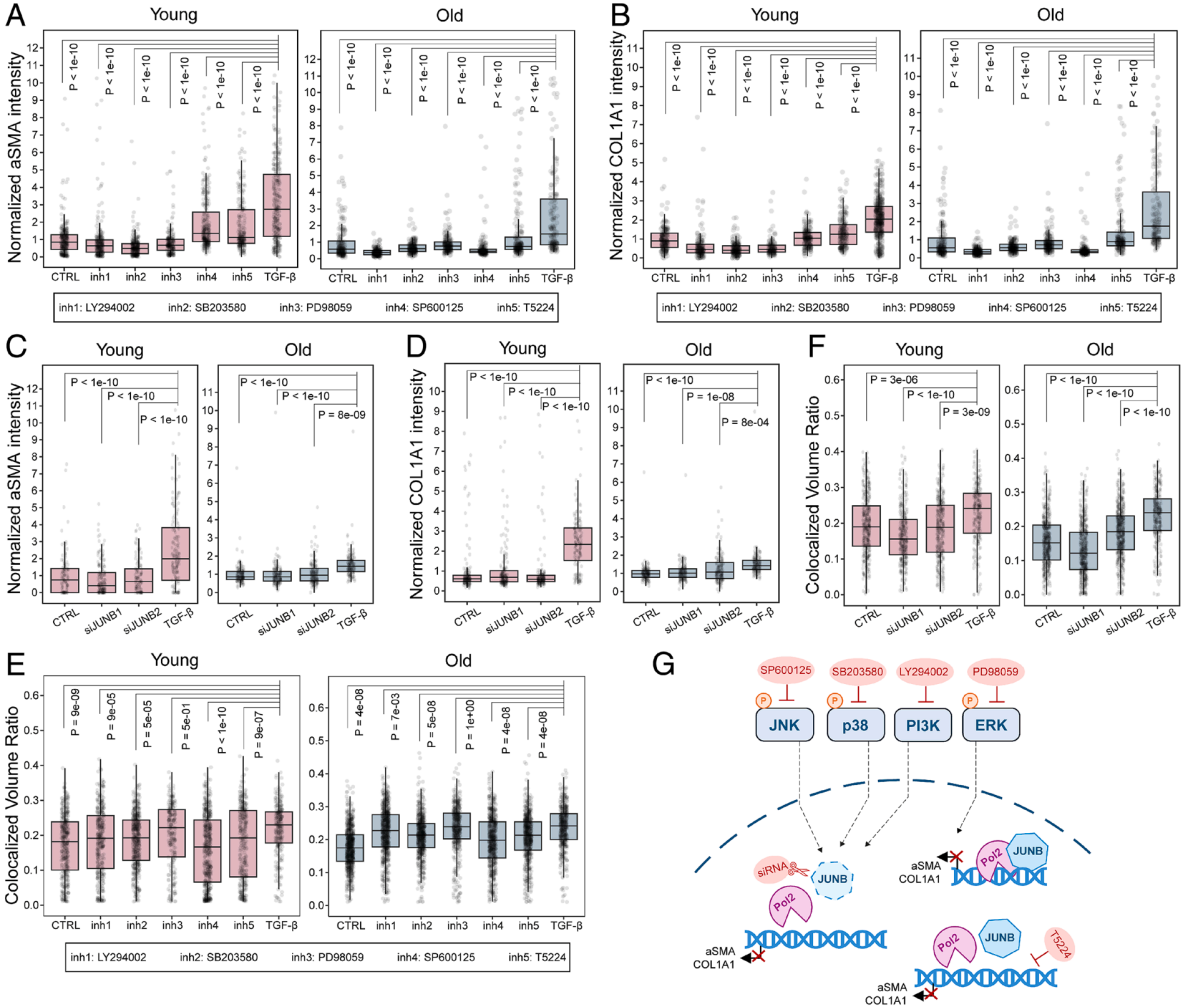
Inhibiting AP-1 suppresses fibroblasts’ responses to TGF-β. (*A* and *B*) Box plots of normalized protein expression markers, (*A*) aSMA and (*B*) COL1A1, of fibroblasts in tensed matrix with or without inhibitor treatments. CTRL: +DMSO/−TGF-β; inh1:+ LY294002/+TGF-β; inh2: +SB203580/+TGF-β; inh3: +PD98059/+TGF-β; inh4: +SP600125/+TGF-β; inh5: +T5224/+TGF-β; TGF-β: +DMSO/+TGF-β. (*C* and *D*) Box plots of normalized protein expression markers, (*C*) aSMA and (*D*) COL1A1, of fibroblasts in tensed matrix with or without siRNA transfection. CTRL: +siNC/−TGF-β; siJUNB11:+ siJUNB1/+TGF-β; siJUNB2: +siJUNB2/+TGF-β; TGF-β: +siNC/+TGF-β (siNC: Negative Control of siRNA). (*E* and *F*) Box plots of JUNB colocalization with RNA polymerase II. Volume ratio: overlapped volume of TF and polymerase normalized by nuclear volume. (*E*) inhibitor treatments; (*F*) siRNA transfection. n ≥ 3, *P*-values are derived from one-way ANOVA and Tukey’s test. (*G*) Schematic of the validation experiments using siRNA and inhibitors.

To confirm the functional role of the AP-1 complex, we performed a comprehensive perturbation study using genetic silencing (siJUNB), direct AP-1 binding inhibition (T-5224), and upstream kinase inhibition (JNK, p38, ERK, PI3K) in young and old fibroblasts (Fig. 6 and *SI Appendix*, Fig. S11). All perturbations significantly suppressed TGF-β-mediated αSMA and COL1A1 induction, demonstrating that AP-1 is an essential driver of fibroblast activation. Mechanistically, costaining with RNA pol II s5 revealed distinct modes of regulation: inhibition of JNK, p38, PI3K, or direct AP-1 binding reduced the nuclear recruitment of JUNB to the transcriptional machinery. In contrast, ERK inhibition preserved the JUNB recruitment, indicating that ERK regulates the postrecruitment potency of the complex rather than its initial genomic occupancy. These findings establish a causal link between the identified kinase-AP-1 regulatory axis. Overall, our findings indicate that AP-1 motifs, in concert with motifs exhibiting age-specific activity, are important drivers of age-dependent differential TGF-β responses.

## Discussion

Our study provides compelling evidence linking cellular mechanics, biochemical signaling, chromatin accessibility, and gene expression. It has been demonstrated that ECM stiffness directs stem cell lineage specification (41), and physical confinement can partially reprogram fibroblasts (42). Separately, biochemical signals like TGF-β are well-known regulators of cellular programs involved in embryonic development, wound healing, tissue homeostasis, and immune homeostasis (23, 43). Building on this, our investigation focused on how the combination of mechanical and biochemical cues affects gene expression during cellular aging. We found that mechanical force alone modulates cell contractility, histone modifications, nuclear features, and gene expression (Fig. 1), consistent with the established effects of mechanical cues on cells. Notably, our results further reveal that matrix tension amplifies cellular responses to TGF-β (Fig. 2). This observation aligns with emerging evidence of synergistic interactions, wherein mechanical inputs prime or enhance biochemical signaling (44–46), underscoring the capacity of cells to actively integrate concurrent stimuli for the precise fine-tuning of transcriptional outputs.

In the context of aging, profound alterations occur in ECM properties, cellular mechanosensing, chromatin organization, and gene expression (17). Our study addresses the interplay between mechanical and biochemical cues by demonstrating that young and old human dermal fibroblasts exhibit distinct transcriptional and functional responses to identical combined mechanical and TGF-β stimulation (Fig. 3). This observed age-dependent modulation of signal integration led us to hypothesize that underlying changes in chromatin accessibility are a primary driver of these divergent outcomes.

To investigate this hypothesis, we employed ATAC-seq, a robust method for mapping genome-wide chromatin accessibility (47, 48). Consistent with existing studies in age-related chromatin accessibility differences (49–51), our ATAC-seq analysis revealed significant differences in chromatin accessibility associated with aging. By combining RNA-seq with ATAC-seq data, we delineated the regulatory networks responsible for these age-specific responses (Figs. 4 and 5). While previous studies have explored aging-related regulatory networks (6, 52), our work uniquely focuses on the synergistic effects of combined mechanical and biochemical stimuli in aging fibroblasts. Our analysis identified the AP-1 TF module as a critical mediator, in concert with TFs exhibiting age-specific activity, for chromatin remodeling. This aligns with recent studies linking AP-1 to age-related chromatin changes and the senescence-associated secretory phenotype (SASP) (53–56). Furthermore, the colocalization of specific TFs, i.e., JUNB, JUND, and HOXB13, with RNA polymerase II lends additional support to the active involvement of these identified networks in the observed transcriptional changes. Importantly, we experimentally validated that genetic silencing or pharmacological disruption of AP-1 complex activity abolishes fibroblast activation, largely by preventing the requisite recruitment of JUNB to the transcriptional machinery. The identification of AP-1 and its associated network as key mediators of these age-specific effects opens up avenues for future research into therapeutic interventions. Further investigation into the precise mechanisms by which AP-1 interacts with the chromatin remodeling machinery and other factors showing age-specific activity in response to combined stimuli will be crucial for developing targeted strategies for healthy aging.

In summary, this study demonstrates that: 1) Mechanical forces and TGF-β signaling synergistically regulate gene expression in human dermal fibroblasts, with matrix tension amplifying TGF-β-induced transcriptional programs in young fibroblasts. 2) Aging significantly alters this synergistic interplay, leading to distinct transcriptional outcomes in old versus young cells subjected to the same combined stimuli, with aged cells showing a blunted or divergent response. 3) Age-differential chromatin accessibility landscapes play a pivotal role in mediating these age-specific responses. 4) The AP-1 TF complex, in conjunction with age-specific interacting TFs, plays a crucial role in remodeling chromatin accessibility and orchestrating the divergent responses to mechanochemical signals during aging. Collectively, these findings highlight the chromatin state as a critical regulatory interface that integrates mechanical and biochemical signals, underscoring how its alteration during aging contributes to modified cellular responsiveness.

## Materials and Methods

Healthy human dermal fibroblasts (GM08401, GM09503, GM01717, and AG04059) from the Coriell Institute were cultured in complete MEM containing 15% FBS, 1% nonessential amino acids, 1% GlutaMAX, and 1% penicillin-streptomycin. To establish 3D cultures, 25,000 cells were embedded in 400 µL of 1 mg/mL collagen within a 21-mm uncoated coverslip-bottom dish (Ibidi) and polymerized at 37 °C for 1.5 h. To model distinct mechanical environments, tensed conditions were created by anchoring the gel periphery with a glass ring (Schmizo AG), whereas unanchored gels served as relaxed conditions. The 3D cultures were maintained for 24 h, serum-starved for 24 h, and then treated with or without 10 ng/mL TGF-β in serum-free medium for an additional 24 h before harvesting. All subsequent functional and molecular experiments were performed using these 3D-embedded fibroblasts subjected to the defined tension states and TGF-β treatments. Detailed protocols for cell culture, cell treatments, cell viability, siRNA transfection, inhibitor treatments, immunostaining, and quantitative image analysis (gel contraction, fluorescence, nuclear features) are provided in the *SI Appendix*, *Materials and Methods*. In addition, detailed procedures for RNA-seq and ATAC-seq, sequencing data analysis, heatmap score calculation, statistical methods, and data availability are also given in *SI Appendix*, *Materials and Methods*.

## Supplementary Material

Appendix 01 (PDF)

Dataset S01 (XLSX)

Dataset S02 (XLSX)

Dataset S03 (XLSX)

Dataset S04 (XLSX)

Dataset S05 (XLSX)

## Data Availability

Sequencing data have been deposited in GEO (GSE300230 (57) and GSE300231 (58)).

## References

[1] S. J. Altschuler, L. F. Wu, Cellular heterogeneity: When do differences make a difference? Cell **141**, 559–563 (2010). 20478246 10.1016/j.cell.2010.04.033 PMC2918286

[2] B. Snijder, L. Pelkmans, Origins of regulated cell-to-cell variability. Nat. Rev. Mol. Cell Biol. **12**, 119–125 (2011). 21224886 10.1038/nrm3044

[3] S. Haas, A. Trumpp, M. D. Milsom, Causes and consequences of hematopoietic stem cell heterogeneity. Cell Stem Cell **22**, 627–638 (2018). 29727678 10.1016/j.stem.2018.04.003

[4] H. Miura, Y. Kondo, M. Matsuda, K. Aoki, Cell-to-cell heterogeneity in p38-mediated cross-inhibition of JNK causes stochastic cell death. Cell Rep. **24**, 2658–2668 (2018). 30184500 10.1016/j.celrep.2018.08.020

[5] C. P. Martinez-Jimenez , Aging increases cell-to-cell transcriptional variability upon immune stimulation. Science **355**, 1433–1436 (2017). 28360329 10.1126/science.aah4115 PMC5405862

[6] C. Jin , Molecular and genetic insights into human ovarian aging from single-nuclei multi-omics analyses. Nat. Aging **5**, 275–290 (2025). 39578560 10.1038/s43587-024-00762-5 PMC11839473

[7] L. A. Trastus, F. d’Adda di Fagagna, The complex interplay between aging and cancer. Nat. Aging **5**, 350–365 (2025). 40038418 10.1038/s43587-025-00827-z PMC7618899

[8] S. S. Khan, B. D. Singer, D. E. Vaughan, Molecular and physiological manifestations and measurement of aging in humans. Aging Cell **16**, 624–633 (2017). 28544158 10.1111/acel.12601 PMC5506433

[9] N. Bosco, M. Noti, The aging gut microbiome and its impact on host immunity. Genes Immun. **22**, 289–303 (2021). 33875817 10.1038/s41435-021-00126-8 PMC8054695

[10] Y. R. Lu, X. Tian, D. A. Sinclair, The information theory of aging. Nat. Aging **3**, 1486–1499 (2023). 38102202 10.1038/s43587-023-00527-6

[11] P. Liu , M6A-independent genome-wide METTL3 and METTL14 redistribution drives the senescence-associated secretory phenotype. Nat. Cell Biol. **23**, 355–365 (2021). 33795874 10.1038/s41556-021-00656-3 PMC8035315

[12] S. W. Criscione , Reorganization of chromosome architecture in replicative cellular senescence. Sci. Adv. **2**, e1500882 (2016). 26989773 10.1126/sciadv.1500882 PMC4788486

[13] Y. Zhao , Multiscale 3d genome reorganization during skeletal muscle stem cell lineage progression and aging. Sci. Adv. **9**, eabo1360 (2023). 36800432 10.1126/sciadv.abo1360 PMC9937580

[14] W. Zhang, J. Qu, G.-H. Liu, J. C. I. Belmonte, The ageing epigenome and its rejuvenation. Nat. Rev. Mol. Cell Biol. **21**, 137–150 (2020). 32020082 10.1038/s41580-019-0204-5

[15] P. Cheung , Single-cell chromatin modification profiling reveals increased epigenetic variations with aging. Cell **173**, 1385–1397.e14 (2018). 29706550 10.1016/j.cell.2018.03.079 PMC5984186

[16] F. Ma , Three-dimensional chromatin reorganization regulates B cell development during ageing. Nat. Cell Biol. **26**, 991–1002 (2024). 38866970 10.1038/s41556-024-01424-9 PMC11178499

[17] G. Kroemer , From geroscience to precision geromedicine: Understanding and managing aging. Cell **188**, 2043–2062 (2025). 40250404 10.1016/j.cell.2025.03.011 PMC12037106

[18] N. Jain, K. V. Iyer, A. Kumar, G. V. Shivashankar, Cell geometric constraints induce modular gene-expression patterns via redistribution of HDAC3 regulated by actomyosin contractility. Proc. Natl. Acad. Sci. **110**, 11349–11354 (2013). 23798429 10.1073/pnas.1300801110 PMC3710882

[19] A. Mitra , Cell geometry dictates TNFα-induced genome response. Proc. Natl. Acad. Sci. U.S.A. **114**, E3882–E3891 (2017). 28461498 10.1073/pnas.1618007114 PMC5441774

[20] S. Venkatachalapathy, D. S. Jokhun, G. V. Shivashankar, Multivariate analysis reveals activation-primed fibroblast geometric states in engineered 3D tumor microenvironments. Mol. Biol. Cell **31**, 803–812 (2020). 32023167 10.1091/mbc.E19-08-0420 PMC7185960

[21] T. Pekeč , Detecting radio- and chemoresistant cells in 3D cancer co-cultures using chromatin biomarkers. Sci. Rep. **13**, 20662 (2023). 38001169 10.1038/s41598-023-47287-2 PMC10673941

[22] R. Gupta , Regulation of p65 nuclear localization and chromatin states by compressive force. Mol. Biol. Cell **36**, ar37 (2025). 39908115 10.1091/mbc.E23-11-0431 PMC12005105

[23] J. Massagué, D. Sheppard, TGF-β signaling in health and disease. Cell **186**, 4007–4037 (2023). 37714133 10.1016/j.cell.2023.07.036 PMC10772989

[24] Y. Kwon , DNA binding and RAD51 engagement by the BRCA2 C-terminus orchestrate DNA repair and replication fork preservation. Nat. Commun. **14**, 432 (2023). 36702902 10.1038/s41467-023-36211-x PMC9879961

[25] C. Cousu , Germinal center output is sustained by HELLS-dependent DNA-methylation-maintenance in B cells. Nat. Commun. **14**, 5695 (2023). 37709749 10.1038/s41467-023-41317-3 PMC10502085

[26] F. Novelli , Germline BARD1 variants predispose to mesothelioma by impairing DNA repair and calcium signaling. Proc. Natl. Acad. Sci. U.S.A. **121**, e2405231121 (2024). 38990952 10.1073/pnas.2405231121 PMC11260134

[27] J. J. Krais , RNF168-mediated localization of BARD1 recruits the BRCA1-PALB2 complex to DNA damage. Nat. Commun. **12**, 5016 (2021). 34408138 10.1038/s41467-021-25346-4 PMC8373961

[28] D. Yang , Histone methyltransferase Smyd3 is a new regulator for vascular senescence. Aging Cell **19**, e13212 (2020). 32779886 10.1111/acel.13212 PMC7511874

[29] B. K. Tripathi , SRC and ERK cooperatively phosphorylate DLC1 and attenuate its Rho-GAP and tumor suppressor functions. J. Cell Biol. **218**, 3060–3076 (2019). 31308216 10.1083/jcb.201810098 PMC6719442

[30] B. K. Tripathi , Receptor tyrosine kinase activation of RhoA is mediated by AKT phosphorylation of DLC1. J. Cell Biol. **216**, 4255–4270 (2017). 29114068 10.1083/jcb.201703105 PMC5716279

[31] A. Chronopoulos , Syndecan-4 tunes cell mechanics by activating the kindlin-integrin-RhoA pathway. Nat. Mater. **19**, 669–678 (2020). 31907416 10.1038/s41563-019-0567-1 PMC7260055

[32] J. Reimand , Pathway enrichment analysis and visualization of omics data using g:Profiler, GSEA, Cytoscape and EnrichmentMap. Nat. Protoc. **14**, 482–517 (2019). 30664679 10.1038/s41596-018-0103-9 PMC6607905

[33] S. Tanigawa , Jun dimerization protein 2 is a critical component of the Nrf2/MafK complex regulating the response to ROS homeostasis. Cell Death Dis. **4**, e921 (2013). 24232097 10.1038/cddis.2013.448 PMC3847324

[34] J. N. R. Gnanaprakasam , Asparagine restriction enhances CD8+ T cell metabolic fitness and antitumoral functionality through an NRF2-dependent stress response. Nat. Metab. **5**, 1423–1439 (2023). 37550596 10.1038/s42255-023-00856-1 PMC10447245

[35] H.-Y. Jung, J. Yang, Unraveling the TWIST between EMT and cancer stemness. Cell Stem Cell **16**, 1–2 (2015). 25575073 10.1016/j.stem.2014.12.005

[36] A. M. Boutelle, L. D. Attardi, P53 and tumor suppression: It takes a network. Trends Cell Biol. **31**, 298–310 (2021). 33518400 10.1016/j.tcb.2020.12.011 PMC7954925

[37] T. Ohira, S. Nakagawa, J. Takeshita, H. Aburatani, H. Kugoh, PITX1 inhibits the growth and proliferation of melanoma cells through regulation of SOX family genes. Sci. Rep. **11**, 18405 (2021). 34526609 10.1038/s41598-021-97791-6 PMC8443576

[38] X. Ma, L. Xu, E. Mueller, Forkhead box A3 mediates glucocorticoid receptor function in adipose tissue. Proc. Natl. Acad. Sci. U.S.A. **113**, 3377–3382 (2016). 26957608 10.1073/pnas.1601281113 PMC4812768

[39] C. Liu , FOXA3 induction under endoplasmic reticulum stress contributes to non-alcoholic fatty liver disease. J. Hepatol. **75**, 150–162 (2021). 33548387 10.1016/j.jhep.2021.01.042

[40] I. Berest , Quantification of differential transcription factor activity and multiomics-based classification into activators and repressors: DiffTF. Cell Rep. **29**, 3147–3159.e12 (2019). 31801079 10.1016/j.celrep.2019.10.106

[41] A. J. Engler, S. Sen, H. L. Sweeney, D. E. Discher, Matrix elasticity directs stem cell lineage specification. Cell **126**, 677–689 (2006). 16923388 10.1016/j.cell.2006.06.044

[42] B. Roy , Fibroblast rejuvenation by mechanical reprogramming and redifferentiation. Proc. Natl. Acad. Sci. U.S.A. **117**, 10131–10141 (2020). 32350144 10.1073/pnas.1911497117 PMC7229653

[43] J. Massagué, TGFβ signalling in context. Nat. Rev. Mol. Cell Biol. **13**, 616–630 (2012). 22992590 10.1038/nrm3434 PMC4027049

[44] A. Totaro, T. Panciera, S. Piccolo, YAP/TAZ upstream signals and downstream responses. Nat. Cell Biol. **20**, 888–899 (2018). 30050119 10.1038/s41556-018-0142-z PMC6186418

[45] D. L. Jones , Mechanoepigenetic regulation of extracellular matrix homeostasis via Yap and Taz. Proc. Natl. Acad. Sci. U.S.A. **120**, e2211947120 (2023). 37216538 10.1073/pnas.2211947120 PMC10235980

[46] K. Kizhatil , FYN regulates aqueous humor outflow and IOP through the phosphorylation of VE-cadherin. Nat. Commun. **16**, 51 (2025). 39746990 10.1038/s41467-024-55232-8 PMC11696269

[47] J. D. Buenrostro, P. G. Giresi, L. C. Zaba, H. Y. Chang, W. J. Greenleaf, Transposition of native chromatin for fast and sensitive epigenomic profiling of open chromatin, DNA-binding proteins and nucleosome position. Nat. Methods **10**, 1213–1218 (2013). 24097267 10.1038/nmeth.2688 PMC3959825

[48] F. C. Grandi, H. Modi, L. Kampman, M. R. Corces, Chromatin accessibility profiling by ATAC-seq. Nat. Protoc. **17**, 1518–1552 (2022). 35478247 10.1038/s41596-022-00692-9 PMC9189070

[49] N. Itokawa , Epigenetic traits inscribed in chromatin accessibility in aged hematopoietic stem cells. Nat. Commun. **13**, 2691 (2022). 35577813 10.1038/s41467-022-30440-2 PMC9110722

[50] R. W. Yeo , Chromatin accessibility dynamics of neurogenic niche cells reveal defects in neural stem cell adhesion and migration during aging. Nat. Aging **3**, 866–893 (2023). 37443352 10.1038/s43587-023-00449-3 PMC10353944

[51] J. Wang , ATAC-seq analysis reveals a widespread decrease of chromatin accessibility in age-related macular degeneration. Nat. Commun. **9**, 1364 (2018). 29636475 10.1038/s41467-018-03856-y PMC5893535

[52] J. M. Braunger, L. V. Cammarata, T. R. Sornapudi, C. Uhler, G. V. Shivashankar, Transcriptional changes are tightly coupled to chromatin reorganization during cellular aging. Aging Cell **23**, e14056 (2024). 38062919 10.1111/acel.14056 PMC10928569

[53] R. Patrick , The activity of early-life gene regulatory elements is hijacked in aging through pervasive AP-1-linked chromatin opening. Cell Metab. **36**, 1858–1881.e23 (2024). 38959897 10.1016/j.cmet.2024.06.006

[54] R. I. Martínez-Zamudio , AP-1 imprints a reversible transcriptional programme of senescent cells. Nat. Cell Biol. **22**, 842–855 (2020). 32514071 10.1038/s41556-020-0529-5 PMC7899185

[55] C. Zhang , ATF3 drives senescence by reconstructing accessible chromatin profiles. Aging Cell **20**, e13315 (2021). 33539668 10.1111/acel.13315 PMC7963335

[56] Y. Wang, L. Liu, Y. Song, X. Yu, H. Deng, Unveiling E2F4, TEAD1 and AP-1 as regulatory transcription factors of the replicative senescence program by multi-omics analysis. Protein Cell **13**, 742–759 (2022). 35023014 10.1007/s13238-021-00894-z PMC9233726

[57] Y. Liao, L. Yuan, T. R. Sornapudi, G. V. Shivashankar, Age-related gene expression alteration responding to mechanical force and TGFβ signaling. https://www.ncbi.nlm.nih.gov/geo/query/acc.cgi?acc=GSE300230. Deposited 20 June 2025.

[58] Y. Liao, L. Yuan, T. R. Sornapud, G. V. Shivashankar, Age-related gene expression alteration responding to mechanical force and TGFβ signaling [ATAC-seq]. https://www.ncbi.nlm.nih.gov/geo/query/acc.cgi?acc=GSE300231. Deposited 20 June 2025.

