# Supplementary material for: Chromatin accessibility regulates age-dependent nuclear mechanotransduction
Source: Proc Natl Acad Sci U S A. 2026 Mar 26;123(13):e2522217123. doi: 10.1073/pnas.2522217123 (PMC13037945; doi:10.1073/pnas.2522217123)
Supplement: Supplementary file 1 — Appendix 01 (PDF) [file pnas.2522217123.sapp.pdf]

PNAS

Supporting Information for

Chromatin Accessibility Regulates Age-Dependent Nuclear Mechanotransduction

Yawen Liao [1,2], Luezhen Yuan [3], Trinadha Rao Sornapudi [1], Max Land [3,4], Rajshikhar Gupta [3], G.V. Shivashankar [1,2,5]

1 Paul Scherrer Institute, Forschungsstrasse 111, Villigen 5232, Switzerland
2 ETH Zurich, Raemisstrasse 101, Zurich 8092, Switzerland
3 Broad Institute of MIT and Harvard, 415 Main Street, Cambridge, MA 02142, USA
4 Massachusetts Institute of Technology, 77 Massachusetts Avenue, Cambridge, MA 02139, USA
5 Corresponding author

Address correspondence to: G.V. Shivashankar Email: gshivasha@ethz.ch

This PDF file includes:

Limitations of the study
Materials and Methods
Figures S1 to S12
Legends for Datasets S1 to S5
SI Reference

Other supporting materials for this manuscript include the following:

Datasets S1 to S5

Limitations of the study

Our *in vitro* model, while useful for examining the role of chromatin, simplifies the more complex *in vivo* environment that features diverse cell interactions, a dynamic matrix, and intercellular signaling. The use of bulk RNA-seq and ATAC-seq provides population-averaged data, lacking paired single-cell resolution and potentially masking cellular heterogeneity; more granular techniques like single-cell omics could offer further detail. Additionally, while we demonstrate strong correlations, establishing direct causality between the stimuli, chromatin alterations, and gene expression requires further genome-wide interventional studies, particularly regarding specific AP-1 subunit networks, to elucidate their precise roles. Despite these constraints, our *in vitro* approach offers a valuable window into this complex system. Future work incorporating single-cell multi-omics will aim to address these limitations and more comprehensively capture the heterogeneity of cellular responses to combined mechanochemical signals during aging.

Materials and Methods

Human primary fibroblasts culture and stimulation
Healthy human dermal fibroblast cells, GM08401 (75 years old, Young), GM09503 (10 years old, Old), GM01717 (39 years old), and AG04059 (96 years old) were obtained from the NIGMS Human Genetic Cell Repository at the Coriell Institute for Medical Research. Human dermal fibroblast cells were cultured in complete medium consisting of Minimum Essential Medium (MEM 11090-081; Gibco) supplemented with 15% (vol/vol) heat-inactivated fetal bovine serum (FBS 16141079; Gibco), 1% nonessential amino acids (11140-035; Gibco), 1% GlutaMAX, and 1% penicillin-streptomycin (392-0406; VWR). For each condition, 25,000 cells were mixed with 400 µL of a 1 mg/mL collagen matrix and cast within the observation area (21 mm diameter) of an uncoated coverslip-bottom dish (Ibidi). After polymerization at 37°C in a CO_2 incubator for 1.5 hours, a glass ring (Ø 20/15 with height 2 mm, Schmizo AG) was placed on top of the collagen gel to cover the gel periphery for the tensed conditions, whereas no ring was applied for the relaxed conditions. Samples were collected under both conditions at designated time points for further experiments. Fibroblasts embedded in the 3D collagen gel, with or without tension, were cultured in complete medium for 24 hours, followed by starvation in serum-free medium for 24 hours. Subsequently, cells were treated with 10 ng/mL TGF-β in serum-free medium for an additional 24 hours. Samples with or without TGF-β treatment were collected for further analyses.

RNA-Seq Sample Preparation and Analysis
Fibroblasts embedded in 3D collagen I gels were stabilized using RNAlater Stabilization Solution (Thermo Fisher Scientific) for 2 minutes, followed by mechanical disruption with a 1.5 mL pestle (Wilmad LabGlass) to facilitate RNA isolation. Total RNA was extracted using the RNeasy Plus Micro Kit (Qiagen). mRNA libraries were prepared using the Illumina Stranded protocol and sequenced on a HiSeq 2000 platform at the Genomics Facility Basel. RNA-seq experiments included four conditions for Tension-stimulus experiments: ON (old fibroblasts without tension), OR (old fibroblasts with tension), YN (young fibroblasts without tension), and YR (young fibroblasts with tension). Additionally, eight conditions were analyzed for Tension/TGF-β stimulation experiments: ONC (old fibroblasts without tension or TGF-β), ONT (old fibroblasts without tension, with TGF-β), ORC (old fibroblasts with tension, without TGF-β), ORT (old fibroblasts with tension and TGF-β), YNC (young fibroblasts without tension or TGF-β), YNT (young fibroblasts without tension, with TGF-β), YRC (young fibroblasts with tension, without TGF-β), and YRT (young fibroblasts with tension and TGF-β). Each condition included two biological replicates of pair-end sequencing, with each replicate sequenced across four technical replicates on different lanes. Sequence reads were aligned using the STAR sequence alignment tool (version 2.7.11b). Specifically, genome indexes were first generated from the Homo sapiens GRCh38 reference genome (obtained from Ensembl) along with genome annotation GTF file (from Ensembl release 112) using the STAR -runMode genomeGenerate --sjdbOverhang 37. Each biological sample (containing 8 fastq file from pair-end sequencing and four technical replicates) was aligned using STAR --outSAMtype BAM SortedByCoordinate --outSAMunmapped Within --outSAMattributes Standard --quantMode GeneCounts --outFilterScoreMinOverLread 0 --outFilterMatchNminOverLread 0. Differential expression (DE) analysis was done using DESeq2 (version 1.44.0). Significant DE genes were called using adj.p value <0.01, |log2FoldChange| > 1. Enrichment pathway network was created based on the previously described method(1) with node cutoff FDR 0.001 (for enriched pathways of upregulated genes) or 0.01 (for enriched pathways of downregulated genes), Jaccard Overlap Combined cutoff (edge cutoff) 0.375 in Cytoscape (version 3.10.2).

ATAC-Seq Sample Preparation and Analysis
Fibroblasts cultured in 3D collagen I gels were treated with collagenase for 20 minutes prior to DNA library preparation using the ATAC-Seq Kit (Active Motif). Sequencing was performed on an Element Aviti platform at the Functional Genomics Center Zurich. ATAC-seq experiments included four conditions: ORC (old fibroblasts with tension, without TGF-β), ORT (old fibroblasts with tension and TGF-β), YRC (young fibroblasts with tension, without TGF-β), and YRT (young fibroblasts with tension and TGF-β). Each condition included two biological replicates. Reads were aligned, filtered for duplicates and mitochondrial reads, and assessed for quality control metrics using the publicly available ENCODE ATAC-seq pipeline (version 2.2.3; https://github.com/ENCODE-DCC/atac-seq-pipeline) implemented in SUSHI platform(2). Briefly, fastq files were trimmed based on adapter sequences by fastp --trim_front1 4 --trim_tail1 0 --average_qual 20 --max_len1 0 --max_len2 0 --trim_poly_x --poly_x_min_len 10 --length_required 30. Bowtie2 was used to align reads to the GRCh38.p13 reference genome with default parameters. Resulting BAM files were filtered to remove PCR duplicates

using Picard (version 2.22.8). Mitochondrial reads were removed. ATAC shifts were corrected using alignmentSieve --ATACshift. Peaks were called using macs2 callpeak –bw 200 --keep-dup all --extsize 200 --binSize 10.
Macs2 identified peaks from two biological replicates were initially combined using intersection (consensus_intersection). Combined peaks from different biological conditions were further merged using union (consensus_union). Counts tables of reads per peak (within either consensus_intersection or consensus_union) were generated using bam file (from bowtie2) for each sample and pysam package. The counts table for consensus_intersection was used for calculating the Pearson correlation coefficient (PCC) with the corresponding annotated gene expression from RNA-seq data. All counts were converted to log2(RPM + 1) before calculating PCC (RPM stands for reads per million). For peaks within 20 kilobases upstream of TSS, the one with the highest PCC for each gene was used in the RNA-seq and ATAC-seq side-by-side heatmap plot. The counts table for consensus_union was used for differential accessible peak analysis. Peaks were annotated by ChIPseeker (1.40.0) R package using default TxDb.Hsapiens.UCSC.hg38.knownGene. Transcription factor (TF) binding sites enrichment analysis for differential accessible peaks was done using HOMER findMotifsGenome (v5.1). TF-target heatmaps were generated after annotating peaks via HOMER annotatePeaks. diffTF (V1.9) was used to calculate TFs activity and classify TFs as activators or repressors by integrative analysis of RNA-seq and ATAC-seq data with default parameters as described previously(3). TF's regulatory network was built by integrating PPI score from STRING (version 12.0) and absolute median Pearson correlation values from diffTF results in Cytoscape (version 3.10.2).

Score calculation
The match ratio shown in Figures 4 and S7 was calculated as follows. First, we identified the number of DE genes in specific pairwise comparisons that were also bound by motifs enriched in a certain group of DACRs. Then, this number was divided by the total number of DE genes. To normalize this ratio, we divided by the total number of motif-binding genes from the DACR group. For the weight score shown in Figures 5, S8, and S9, we first calculated the mean values of gene log2FoldChange for two pairwise comparisons. We scaled these mean values by dividing them by the maximum mean. Additionally, we scaled the Fold Enrichment of the motif by dividing it by the maximum. Then we multiplied the scaled gene log2FoldChange mean values by the scaled motif Fold Enrichment to obtain the weight scores.

Acquisition of gene set
The Smad2/3/4 target gene set used in Figure S3I was obtained from hTFtarget database. The TFs set used in Figure S3J was obtained from HOMER Motif Database. The mechanical stimulus related gene set used in Figure S3K was obtained from GO with terms GO:0009612, GO:0071260, GO:0050982, GO:0008381, and GO:0098782. The TGF-β response pathway gene set was obtained by combining TGF-β pathway genes from GO (GO:0007179, GO:0071559), KEGG, WiKiPathways, Panther, and Reactome databases. The ECM regulation gene set was obtained from terms that contain keywords “extracellular matrix” or “collagen” in GO and Reactomes.

Immunostaining
Fibroblasts embedded in collagen I gels were fixed with 4% paraformaldehyde (Sigma-Aldrich) in 1x PBS buffer (pH 7.4) for 25 minutes, followed by three washes with 1x PBS containing 100 mM glycine (15 minutes per wash). Cells were permeabilized with 0.5% Triton X-100 (Sigma-Aldrich) in 1x PBS for 20 minutes, followed by three washes with 1x PBS–glycine buffer (15 minutes per wash). Blocking was performed using 10% goat serum (Thermo Fisher Scientific) in immunofluorescence (IF) wash buffer (1x PBS with 0.2% Triton X-100 and 0.2% Tween-20) for 3 hours at room temperature. Primary antibody incubation was carried out overnight at 4°C using antibodies diluted in the blocking buffer, followed by three washes with IF wash buffer (15 minutes per wash). Cells were then incubated with fluorescent-labeled secondary antibodies diluted in 5% goat serum in IF wash buffer for 3 hours at room temperature. The primary antibodies used are listed below: Ki67 (1:6400; CST 9449); pMLC (1:500; CST 95777); H3K9me3 (1:250; Abcam ab176916); H3K27me3 (1:800; CST 9733); H3K27ac (1:200; CST 8173); HP1a (1:250; CST 2616); pSMAD2 (1:500; CST 18338); pSMAD3 (1:200; CST 9520); aSMA (1:1000; Abcam ab7817); COL1A1 (1:700; Abcam ab138492); FN1 (1:200; Abcam ab2413); JUNB (1:200; CST 3753); JUND (1:200; CST 5000); HOXB13 (1:100; CST 90944); RNA ploymerase II S5 (1:700; Abcam ab252852). Secondary antibodies: Alexa Fluor Plus 555 donkey anti-mouse IgG secondary antibody (Invitrogen A32773); Alexa Fluor Plus 647 goat anti-rabbit IgG secondary antibody (Invitrogen A32733); Alexa Fluor Plus 647 donkey anti-rat IgG secondary antibody (Invitrogen A48272). Nuclei and filamentous actin were stained with NucBlue Live Ready Probes (R37605, Thermo Fisher Scientific) at a concentration of two drops per milliliter in 1x PBS for 3 hours at room temperature or overnight at 4°C. Filamentous actin was labeled using ActinGreen Ready Probes (R37110, Thermo Fisher Scientific) at a concentration of two drops per milliliter in 1x PBS, incubated for 3 hours at room temperature or overnight at 4°C.

Cell viability test via DRAQ7
Fibroblasts were cultured in 3D collagen I gels for 24 hours, followed by two washes with 1x PBS. A 1ml solution of DRAQ7 (1:200 in 1x PBS; Biolegend 424001) and NucBlue (two drops per milliliter of 1x PBS) staining buffer was added per gel and incubated at 37°C in a CO_2 incubator for 1 hour. Positive control samples were treated with Staurosporine (1 μM, CST 9953) for 24 hours before staining.

siRNA Transfection and Inhibitor Treatments

Human dermal fibroblasts were subjected to transient genetic knockdown in 2D culture prior to 3D embedding. Cells were seeded at 70–80% confluency in 6-well plates and transfected with either JUNB-specific siRNA or a non-targeting control siRNA (siNC) using Lipofectamine 3000 (Invitrogen) in Opti-MEM medium, following the manufacturer’s instructions. At 24 hours post-transfection, cells were trypsinized and seeded into 3D collagen type I gels. Embedded cells were subsequently serum-starved for 24 hours and stimulated with 10ng/mL TGF-β1 for a final 24-hour period. For pathway inhibition studies, fibroblasts were treated with inhibitors before TGF-β1 treatment. Human dermal fibroblasts were subjected to transient genetic knockdown in 2D culture prior to 3D embedding. Cells were seeded at 70–80% confluency in 6-well plates and transfected with either JUNB-specific siRNA (IDT, TriFECTa DsiRNA Kit hs.Ri.JUNB.13. siJUNB1: SEQ1, rGrArArArCrArCrGrCrArCrUrUrArGrUrCrUrCrUrArArAGA; SEQ2, rUrCrUrUrUrArGrArGrArCrUrArArGrUrGrCrGrUrGrUrUrUrCrUrU. siJUNB2: SEQ1, rGrArArCrGrCrCrUrGrArUrUrGrUrCrCrCrCrArArCrArGCA; SEQ2, rUrGrCrUrGrUrUrGrGrGrGrArCrArArUrCrArGrGrCrGrUrUrCrCrA.) or a non-targeting control siRNA (siNC) using Lipofectamine 3000 (Invitrogen) in Opti-MEM medium, following the manufacturer’s instructions. At 24 hours post-transfection, cells were trypsinized and seeded into 3D collagen type I gels. Embedded cells were subsequently serum-starved for 24 hours and stimulated with 10ng/mL TGF-β1 for a final 24-hour period.

For pathway inhibition studies, human dermal fibroblasts were seeded in 3D collagen type I gels and allowed to stabilize for 24 hours in complete medium. Cells were then serum-starved in serum-free medium for 24 hours. Specific signaling pathways were targeted using the following small molecule inhibitors: T-5224 (Merck HY-12270, 10μM), SP600125 (Merck HY-12041, 10μM), SB203580 (Merck HY-10256, 10μM), PD98059 (Merck HY-12028, 10μM), and LY294002 (Merck HY-10108A, 10μM). Inhibitors were prepared in DMSO and added to fresh serum-free medium for a 1-hour pre-incubation period prior to stimulation. Following pre-incubation, cells were treated with 10ng/mL TGF-β1 in the presence of the inhibitors for an additional 24 hours. Control groups were treated with an equal volume of DMSO vehicle.

Image Acquisition and Analysis

Fluorescent images of fibroblasts embedded in 3D collagen I gels were acquired using either a Nikon A1R laser-scanning, a Nikon spinning disk confocal microscope, or a Nikon DeepSIM (Nikon Instruments Inc.). Nuclear features were analyzed based on stained nuclei images as described in previous work(4). Fluorescent images of fibroblasts embedded in 3D collagen I gels were acquired using either a Nikon A1R laser-scanning, a Nikon spinning disk confocal microscope, or a Nikon DeepSIM (Nikon Instruments Inc.) under either 20× magnification (Plan Apo 20× extra long working distance, numerical aperture [NA] 0.8), 40× magnification (Plan Apo λ 40x or 1.25-NA silicone immersion objective), with identical acquisition settings across samples. In the Z dimension, each 3D gel was scanned to a depth of up to 40 μm, with a step size ranging from 1 to 2 μm. Confocal images were captured with an XY optical resolution of 0.86, 0.43, or 0.24 μm, depending on the objective and imaging settings. Representative images of TFs colocalization with RNA polymerase II were captured using Nikon DeepSIM under 60x magnification (PLAN APO λD 60x OIL OFN25 DIC N2) with 0.06 μm per pixel resolution. For gel contraction assays, images of collagen gels at different time points during cell growth were captured with a mobile camera at a fixed magnification. Fluorescence intensities for each protein were measured in their respective channels using custom-written scripts in R and Python. After nucleus image feature extraction, each feature was normalized using (value-mean+2*standard deviation)/(4*standard deviation) and then capped at 0-1 range. Features were reviewed manually, one by one, to remove those exhibiting a large batch effect, reducing them to 54 features. Scanpy package was then used as a standard single cell analysis pipeline, including PCA (scanpy.pp.pca), batch correction using harmony (scanpy.external.pp.harmony_integrate), finding neighbors (scanpy.pp.neighbors, number of neighbors: k=15, use_rep='X_pca_harmony'), UMAP projection (scanpy.tl.umap), and Leiden clustering (scanpy.tl.leiden, resolution=0.3). Default parameters were used for Scanpy pipelines that were not otherwise described. Top features distinguishing different Leiden clusters were calculated using rank_genes_groups_dotplot in scanpy. The descriptive label for each Leiden cluster was made based on these top distinguishing features.

Statistical Analysis

For box plots in Figures S1, S2 and S11, the box limits represent the 25th to 75th percentiles, with all data points shown. For violin plots in Figures 2, 5, and S1, bar labels indicate the 25th to 75th percentiles, with all data points shown. Data in Figures S1B, S1C, and S3D are presented as mean ± SEM. Each experiment was conducted in at least three independent replicates. Statistical significance of mean differences was evaluated using two-sided Unpaired t test, two-sided Wilcoxon tests, one-way ANOVA and Tukey’s multiple comparison test, or Proportion Z-Test, comparing the sample of interest to the corresponding control.

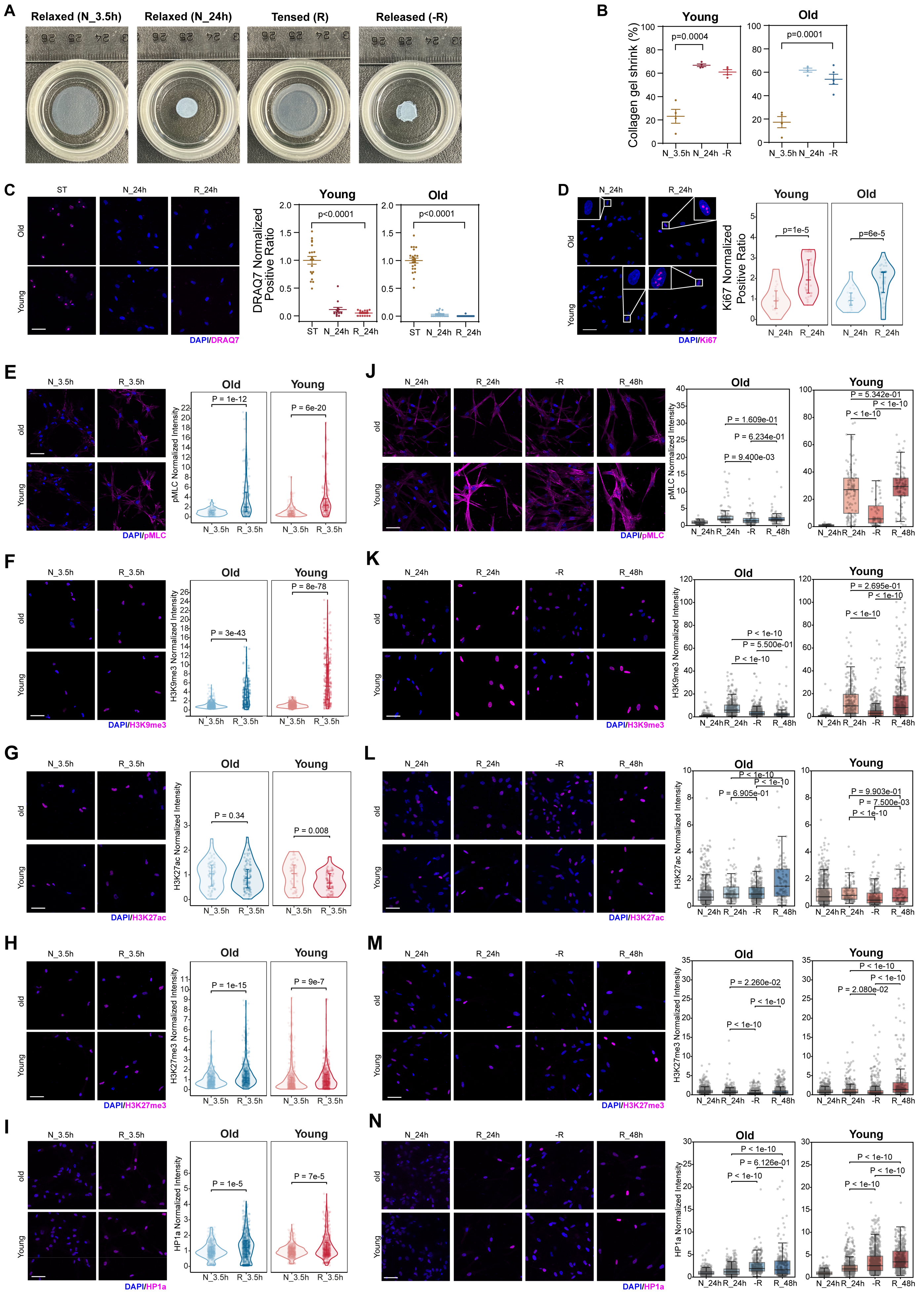
A
Relaxed (N_3.5h)
Relaxed (N_24h)
Tensed (R)
Released (-R)
B
Young
Old
Collagen gel shrink (%)
p=0.0004
p=0.0001
N_3.5h N_24h -R
C
ST
N_24h
R_24h
Old
Young
DAPI/DRAQ7
DRAQ7 Normalized Positive Ratio
p<0.0001
D
Ki67 Normalized Positive Ratio
p=1e-5
p=6e-5
DAPI/Ki67
E
N_3.5h
R_3.5h
pMLC Normalized Intensity
P = 1e-12
P = 6e-20
DAPI/pMLC
J
N_24h
R_24h
-R
R_48h
P = 1.609e-01
P = 6.234e-01
P = 9.400e-03
P = 5.342e-01
P < 1e-10
F
H3K9me3 Normalized Intensity
P = 3e-43
P = 8e-78
DAPI/H3K9me3
K
P = 5.500e-01
P = 2.695e-01
G
H3K27ac Normalized Intensity
P = 0.34
P = 0.008
DAPI/H3K27ac
L
P = 6.905e-01
P = 9.903e-01
P = 7.500e-03
H
H3K27me3 Normalized Intensity
P = 1e-15
P = 9e-7
DAPI/H3K27me3
M
P = 2.260e-02
P = 2.080e-02
I
HP1a Normalized Intensity
P = 1e-5
P = 7e-5
DAPI/HP1a
N
P = 6.126e-01

Figure S1. Collagen gel shrinkage, DRAQ7, and immunostaining results, related to Figure 1
(A) Representative images of Relaxed (3.5h and 24h), Tensed, and Released collagen gels.
(B) Percentage of gel shrinkage in Relaxed and Released conditions calculated based on gel diameter. Data are presented as mean ± SEM. p-values are calculated by the two-sided Unpaired t test, n=3.
(C) DRAQ7 positive ratio indicating cell viability after 24h of tension stimulus. Staurosporine (1 μM, ST) was used to induce cell apoptosis as the positive control. Data was normalized by the mean positive ratio of ST condition. Data are presented as mean ± SEM. p-values are calculated by the two-sided Unpaired t test, n=3. Representative images scale bar: 50μm.
(D) Ki67 positive ratio indicating cell proliferation after 24h of tension stimulus. Data was normalized by the mean positive ratio of Relaxed conditions. p-values are calculated by the two-sided Wilcoxon rank-sum test, n=3. Representative images scale bar: 50μm.
(E-I) Representative images and violin plots of normalized protein markers expression **(E)** pMLC, **(F)** H3K9me3, **(G)** H3K27ac, **(H)** H3K27me3, and **(I)** HP1a with/without tension stimulus for 3.5h. All data points are shown in plots; quartiles indicated by lines. p-values are calculated by the two-sided Wilcoxon rank-sum test, $n \geq 3$. Scale bar: 50μm.
(J-N) Representative images and box plots of normalized protein marker expression **(J)** pMLC, **(K)** H3K9me3, **(L)** H3K27ac, **(M)** H3K27me3, and **(N)** HP1a with/without tension stimulus for long-term (24h and 48h). $n \geq 3$, p-values are calculated by the one-way ANOVA and Tukey's multiple comparison test. Scale bar: 50μm.

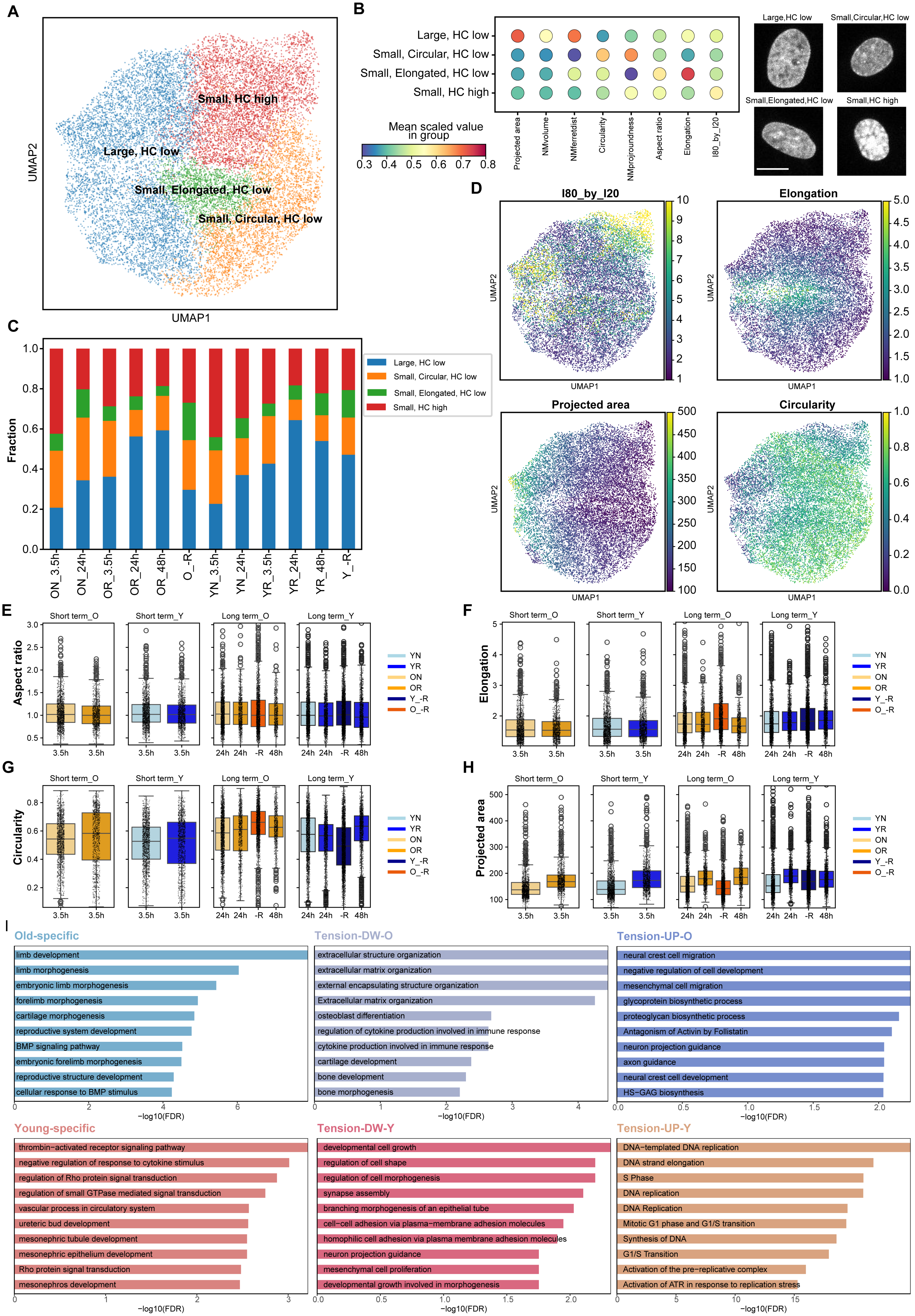
A
UMAP2
UMAP1
Small, HC high
Large, HC low
Small, Elongated, HC low
Small, Circular, HC low
B
Large, HC low
Small, Circular, HC low
Small, Elongated, HC low
Small, HC high
Mean scaled value in group
0.3 0.4 0.5 0.6 0.7 0.8
Projected area
NMvolume
NMferretdist
Circularity
NMprojroundness
Aspect ratio
Elongation
I80_by_I20
Large,HC low
Small,Circular,HC low
Small,Elongated,HC low
Small,HC high
C
Fraction
1.0 0.8 0.6 0.4 0.2 0.0
Large, HC low
Small, Circular, HC low
Small, Elongated, HC low
Small, HC high
ON_3.5h ON_24h OR_3.5h OR_24h OR_48h O_-R YN_3.5h YN_24h YR_3.5h YR_24h YR_48h Y_-R
D
I80_by_I20
Elongation
Projected area
Circularity
UMAP2
UMAP1
E
Aspect ratio
Short term_O
Short term_Y
Long term_O
Long term_Y
3.5h 3.5h 3.5h 3.5h 24h 24h -R 48h 24h 24h -R 48h
YN
YR
ON
OR
Y_-R
O_-R
F
Elongation
G
Circularity
H
Projected area
I
Old-specific
limb development
limb morphogenesis
embryonic limb morphogenesis
forelimb morphogenesis
cartilage morphogenesis
reproductive system development
BMP signaling pathway
embryonic forelimb morphogenesis
reproductive structure development
cellular response to BMP stimulus
-log10(FDR)
Tension-DW-O
extracellular structure organization
extracellular matrix organization
external encapsulating structure organization
Extracellular matrix organization
osteoblast differentiation
regulation of cytokine production involved in immune response
cytokine production involved in immune response
cartilage development
bone development
bone morphogenesis
Tension-UP-O
neural crest cell migration
negative regulation of cell development
mesenchymal cell migration
glycoprotein biosynthetic process
proteoglycan biosynthetic process
Antagonism of Activin by Follistatin
neuron projection guidance
axon guidance
neural crest cell development
HS−GAG biosynthesis
Young-specific
thrombin−activated receptor signaling pathway
negative regulation of response to cytokine stimulus
regulation of Rho protein signal transduction
regulation of small GTPase mediated signal transduction
vascular process in circulatory system
ureteric bud development
mesonephric tubule development
mesonephric epithelium development
Rho protein signal transduction
mesonephros development
Tension-DW-Y
developmental cell growth
regulation of cell shape
regulation of cell morphogenesis
synapse assembly
branching morphogenesis of an epithelial tube
cell−cell adhesion via plasma−membrane adhesion molecules
homophilic cell adhesion via plasma membrane adhesion molecules
neuron projection guidance
mesenchymal cell proliferation
developmental growth involved in morphogenesis
Tension-UP-Y
DNA−templated DNA replication
DNA strand elongation
S Phase
DNA replication
DNA Replication
Mitotic G1 phase and G1/S transition
Synthesis of DNA
G1/S Transition
Activation of the pre−replicative complex
Activation of ATR in response to replication stress

Figure S2 Nuclear features analysis and enriched pathways, related to Figure 1
(A) UMAP showing the Leiden clusters with cluster labels. HC: Heterochromatin content. Each dot represents one nucleus. Data points include all data from the 12 conditions listed in (C).
(B) Dot plot of selected nuclear features displaying the feature variations among Leiden clusters. Color: scaled feature mean value. Representative images of the nucleus in each cluster, scale bar: 10μm.
(C) Bar plots of the Leiden cluster fractions in each condition.
(D) UMAP with selected features highlighted, the scaled mean value is indicated by color.
(E-H) Box plots of **(E)** aspect ratio, **(F)** elongation, **(G)** circularity, and **(H)** projected area.
(I) Bar plots of the top 10 enriched pathways for each DE gene cluster in Figure 1E.

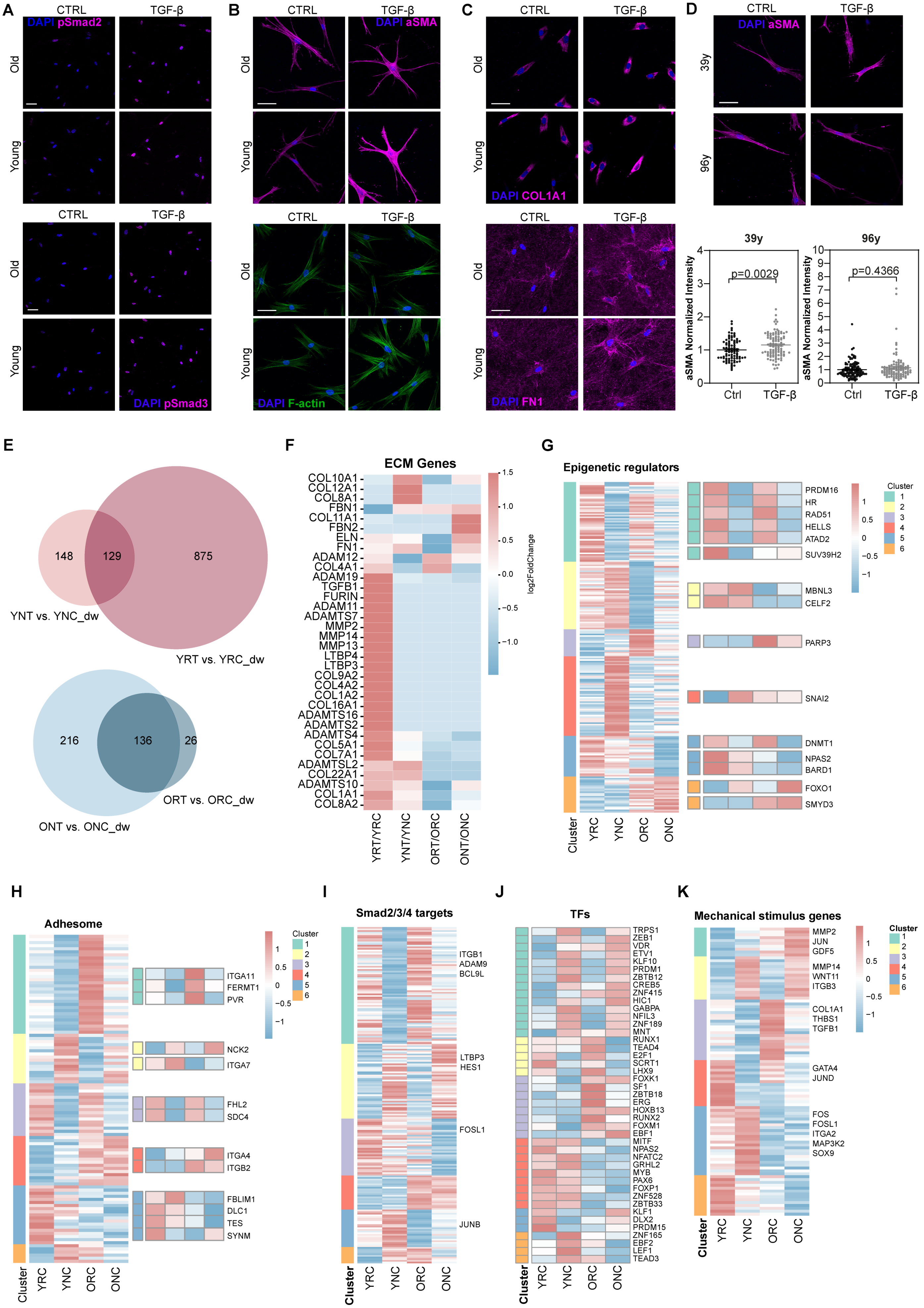
A
CTRL
TGF-β
DAPI pSmad2
Old
Young
DAPI pSmad3
B
DAPI aSMA
DAPI F-actin
C
DAPI COL1A1
DAPI FN1
D
39y
96y
aSMA Normalized Intensity
p=0.0029
p=0.4366
Ctrl
TGF-β
E
148
129
875
YNT vs. YNC_dw
YRT vs. YRC_dw
216
136
26
ONT vs. ONC_dw
ORT vs. ORC_dw
F
ECM Genes
COL10A1
COL12A1
COL8A1
FBN1
COL11A1
FBN2
ELN
FN1
ADAM12
COL4A1
ADAM19
TGFB1
FURIN
ADAM11
ADAMTS7
MMP2
MMP14
MMP13
LTBP4
LTBP3
COL9A2
COL4A2
COL1A2
COL16A1
ADAMTS16
ADAMTS2
ADAMTS4
COL5A1
COL7A1
ADAMTSL2
COL22A1
ADAMTS10
COL1A1
COL8A2
log2FoldChange
YRT/YRC
YNT/YNC
ORT/ORC
ONT/ONC
G
Epigenetic regulators
PRDM16
HR
RAD51
HELLS
ATAD2
SUV39H2
MBNL3
CELF2
PARP3
SNAI2
DNMT1
NPAS2
BARD1
FOXO1
SMYD3
Cluster
YRC
YNC
ORC
ONC
H
Adhesome
ITGA11
FERMT1
PVR
NCK2
ITGA7
FHL2
SDC4
ITGA4
ITGB2
FBLIM1
DLC1
TES
SYNM
I
Smad2/3/4 targets
ITGB1
ADAM9
BCL9L
LTBP3
HES1
FOSL1
JUNB
J
TFs
TRPS1
ZEB1
VDR
ETV1
KLF10
PRDM1
ZBTB12
CREB5
ZNF415
HIC1
GABPA
NFIL3
ZNF189
MNT
RUNX1
TEAD4
E2F1
SCRT1
LHX9
FOXK1
SF1
ZBTB18
ERG
HOXB13
RUNX2
FOXM1
EBF1
MITF
NPAS2
NFATC2
GRHL2
MYB
PAX6
FOXP1
ZNF528
ZBTB33
KLF1
DLX2
PRDM15
ZNF165
EBF2
LEF1
TEAD3
K
Mechanical stimulus genes
MMP2
JUN
GDF5
MMP14
WNT11
ITGB3
COL1A1
THBS1
TGFB1
GATA4
JUND
FOS
FOSL1
ITGA2
MAP3K2
SOX9

Figure S3 Protein immunostaining and DE genes results, related to Figure 2
(A-C) Representative images of immunostained **(A)** pSmad2 and pSmad3, **(B)** aSMA and F-actin, and **(C)** COL1A1 and FN1.
(D) Representative images of immunostained aSMA and dot plots showing normalized expression values of aSMA in fibroblasts (39 years old, 39y; 96 years old, 96y) with or without TGF-β treatment. Data are presented as mean ± SEM. p-values are calculated by the two-sided Wilcoxon rank-sum test, n=3.
(E) Venn diagrams showing overlap of TGF-β-induced downregulated genes (adj.p value <0.01, log2FoldChange < -1) between tensed (R) and relaxed (N) conditions. YRT vs. YRC_dw: downregulated genes in YRT vs. YRC; YNT vs. YNC_dw: downregulated genes in YNT vs. YNC; ORT vs. ORC_dw: downregulated genes in ORT vs. ORC; ONT vs. ONC_dw: downregulated genes in ONT vs. ONC.
(F) Gene expression heatmap of selected ECM-related genes. Values are the scaled log2FoldChange values in each pairwise comparison.
(G-H) Hierarchical clustering of **(G)** epigenetic regulators and **(H)** adhesomes gene expression in conditions with tension without TGF-β. Labeled genes on the right side are the overlapped genes also found in Tension-stimulus RNA-seq data **(Figure 1E)**.
(I) Hierarchical clustering of target genes of Smad2, Smad3, and Smad4 gene expression in conditions with tension without TGF-β. Labeled genes on the right side are selected genes involved in TGF-β signaling.
(J) Hierarchical clustering of TFs gene expression. TFs mainly exhibit age specificity, with high expression in specific age groups.
(K) Hierarchical clustering of mechanical stimulus genes expression. Labeled genes on the right side are selected genes involved in TGF-β signaling.
The values in (G-K) are the scaled $\log_2$(RPM+1) of Tension/TGF-β-stimuli conditions RNA-seq data.

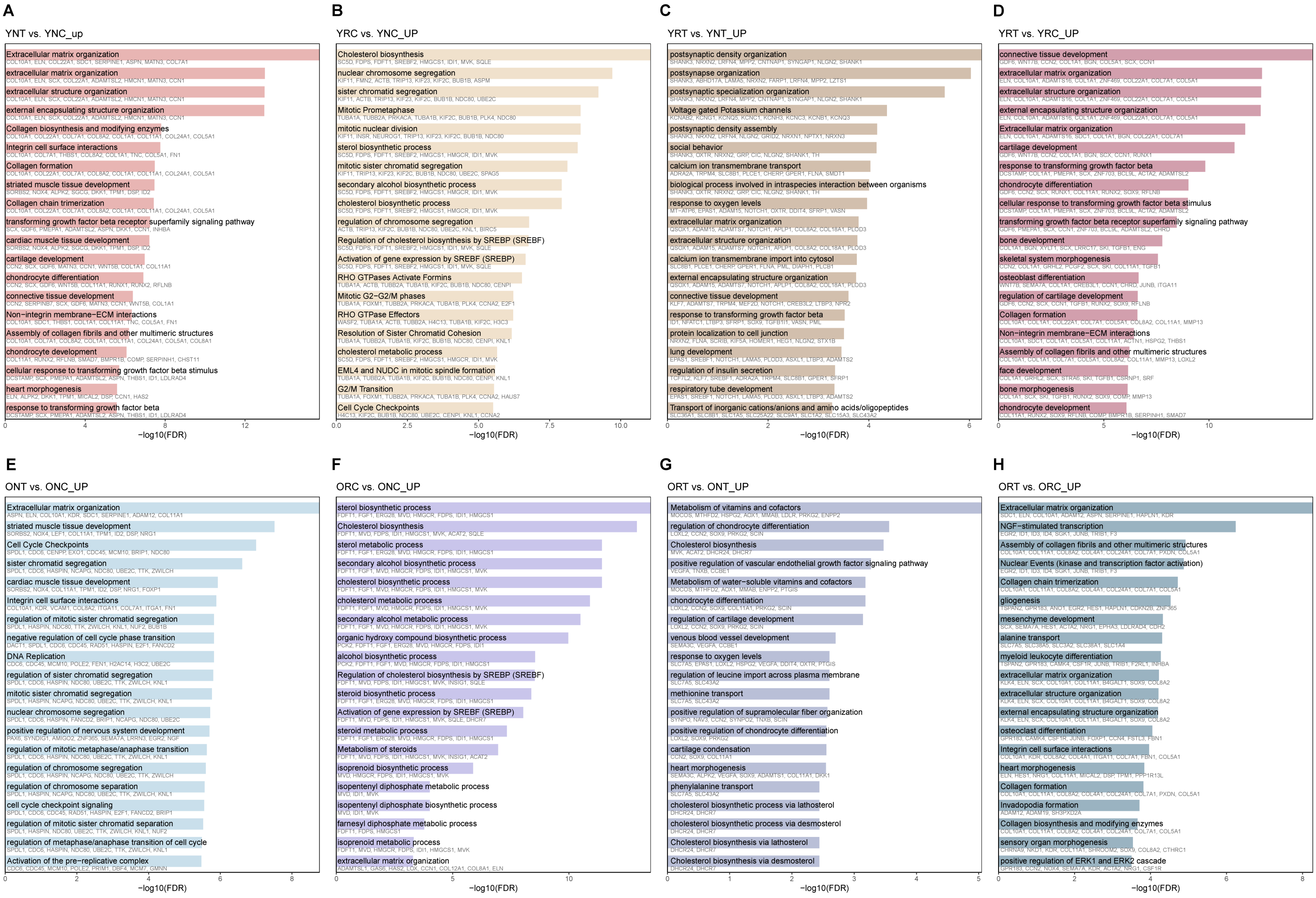

Figure S4 Enriched pathways, related to Figure 2

(A-H) Bar plots of the top 20 enriched pathways with the top 8 upregulated genes listed for each pairwise comparison.

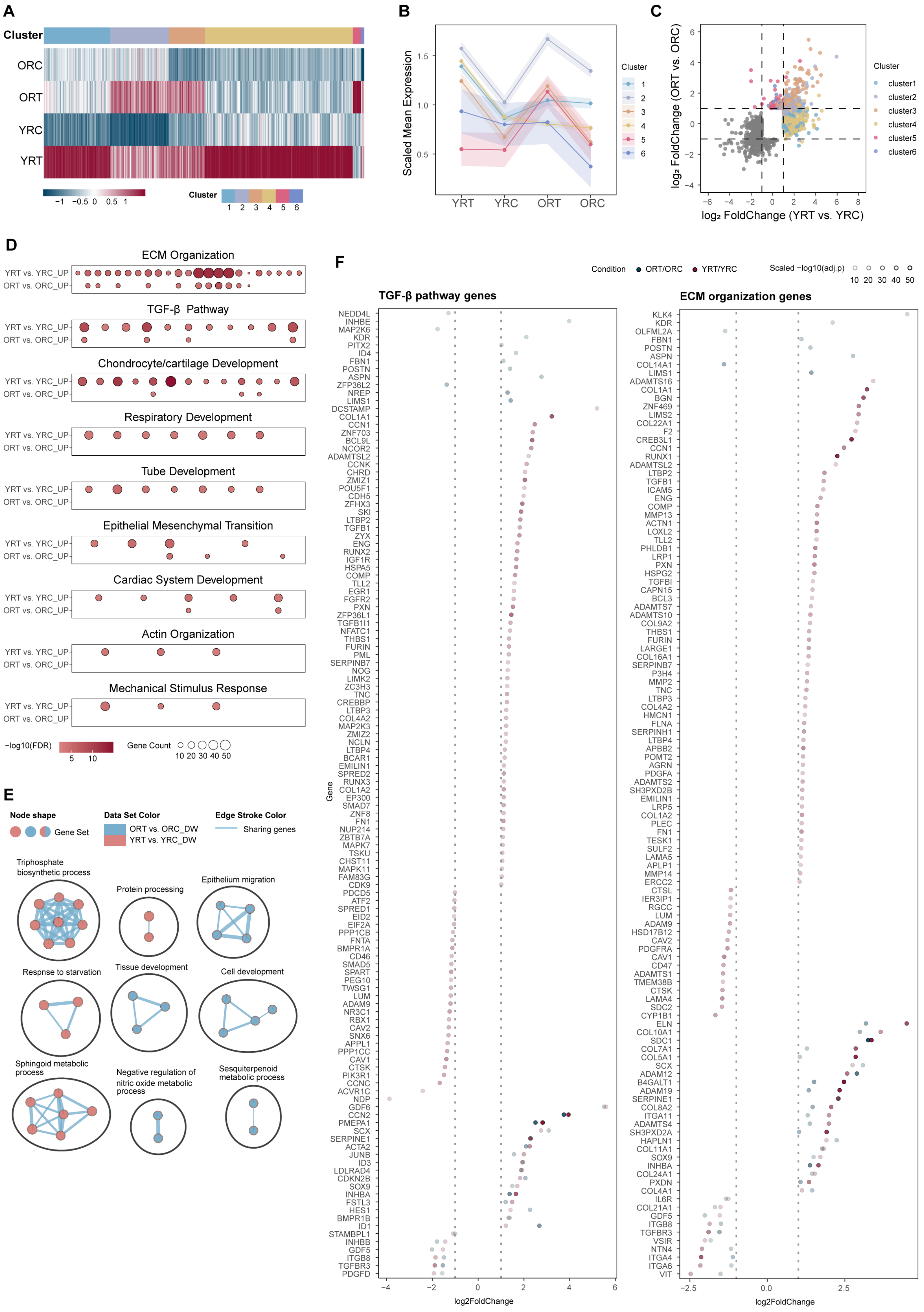

A
Cluster
ORC
ORT
YRC
YRT
−1 −0.5 0 0.5 1
Cluster
1 2 3 4 5 6
B
Scaled Mean Expression
0.5
1.0
1.5
YRT YRC ORT ORC
Cluster
1
2
3
4
5
6
C
log₂ FoldChange (ORT vs. ORC)
log₂ FoldChange (YRT vs. YRC)
Cluster
cluster1
cluster2
cluster3
cluster4
cluster5
cluster6
D
ECM Organization
TGF-β Pathway
Chondrocyte/cartilage Development
Respiratory Development
Tube Development
Epithelial Mesenchymal Transition
Cardiac System Development
Actin Organization
Mechanical Stimulus Response
YRT vs. YRC_UP
ORT vs. ORC_UP
−log10(FDR)
5 10
Gene Count
10 20 30 40 50
E
Node shape
Gene Set
Data Set Color
ORT vs. ORC_DW
YRT vs. YRC_DW
Edge Stroke Color
Sharing genes
Triphosphate biosynthetic process
Protein processing
Epithelium migration
Respnse to starvation
Tissue development
Cell development
Sphingoid metabolic process
Negative regulation of nitric oxide metabolic process
Sesquiterpenoid metabolic process
F
Condition
ORT/ORC
YRT/YRC
Scaled −log10(adj.p)
10 20 30 40 50
TGF-β pathway genes
ECM organization genes
Gene
log2FoldChange
NEDD4L INHBE MAP2K6 KDR PITX2 ID4 FBN1 POSTN ASPN ZFP36L2 NREP LIMS1 DCSTAMP COL1A1 CCN1 ZNF703 BCL9L NCOR2 ADAMTSL2 CCNK CHRD ZMIZ1 POU5F1 CDH5 ZFHX3 SKI LTBP2 TGFB1 ZYX ENG RUNX2 IGF1R HSPA5 COMP TLL2 EGR1 FGFR2 PXN ZFP36L1 TGFB1I1 NFATC1 THBS1 FURIN PML SERPINB7 NOG LIMK2 ZC3H3 TNC CREBBP LTBP3 COL4A2 MAP2K3 ZMIZ2 NCLN LTBP4 BCAR1 EMILIN1 SPRED2 RUNX3 COL1A2 EP300 SMAD7 ZNF8 FN1 NUP214 ZBTB7A MAPK7 TSKU CHST11 MAPK11 FAM83G CDK9 PDCD5 ATF2 SPRED1 EID2 EIF2A PPP1CB FNTA BMPR1A CD46 SMAD5 SPART PEG10 TWSG1 LUM ADAM9 NR3C1 RBX1 CAV2 SNX6 APPL1 PPP1CC CAV1 CTSK PIK3R1 CCNC ACVR1C NDP GDF6 CCN2 PMEPA1 SCX SERPINE1 ACTA2 JUNB ID3 LDLRAD4 CDKN2B SOX9 INHBA FSTL3 HES1 BMPR1B ID1 STAMBPL1 INHBB GDF5 ITGB8 TGFBR3 PDGFD
KLK4 KDR OLFML2A FBN1 POSTN ASPN COL14A1 LIMS1 ADAMTS16 COL1A1 BGN ZNF469 LIMS2 COL22A1 F2 CREB3L1 CCN1 RUNX1 ADAMTSL2 LTBP2 TGFB1 ICAM5 ENG COMP MMP13 ACTN1 LOXL2 TLL2 PHLDB1 LRP1 PXN HSPG2 TGFBI CAPN15 BCL3 ADAMTS7 ADAMTS10 COL9A2 THBS1 FURIN LARGE1 COL16A1 SERPINB7 P3H4 MMP2 TNC LTBP3 COL4A2 HMCN1 FLNA SERPINH1 LTBP4 APBB2 POMT2 AGRN PDGFA ADAMTS2 SH3PXD2B EMILIN1 LRP5 COL1A2 PLEC FN1 TESK1 SULF2 LAMA5 APLP1 MMP14 ERCC2 CTSL IER3IP1 RGCC LUM ADAM9 HSD17B12 CAV2 PDGFRA CAV1 CD47 ADAMTS1 TMEM38B CTSK LAMA4 SDC2 CYP1B1 ELN COL10A1 SDC1 COL7A1 COL5A1 SCX ADAM12 B4GALT1 ADAM19 SERPINE1 COL8A2 ITGA11 ADAMTS4 SH3PXD2A HAPLN1 COL11A1 SOX9 INHBA COL24A1 PXDN COL4A1 IL6R COL21A1 GDF5 ITGB8 TGFBR3 VSIR NTN4 ITGA4 ITGA6 VIT

Figure S5 Gene clusters of upregulated genes in YRT vs. YRC and ORT vs. ORC, enrichment pathway network, and TGF-β response/ ECM regulation pathways gene expression, related to Figure 3
(A) Hierarchical clustering of upregulated genes in YRT vs. YRC and ORT vs. ORC. The values are the scaled log_2(RPM+1).
(B) Line chart of mean scaled log_2(RPM+1) value of genes in each cluster. Shadow: standard error of the mean, SEM.
(C) Scatter plot of upregulated genes in young (x-axis) and old (y-axis) cells labeled in clusters. Grey: downregulated genes.
(D) Dot plots showing the enriched pathways of upregulated genes of YRT vs. YRC and ORT vs. ORC, respectively. Each dot indicates one pathway term with color representing -log_{10}FDR and size representing enriched gene numbers. Pathway clusters are based on the pathway enrichment network (Figure 3D, Datasets S1 and S2). The selected pathway clusters are displayed.
(E) Enrichment pathway network based on downregulated genes in YRT vs. YRC and ORT vs. ORC comparisons. Each dot represents an enriched pathway term; dot in colors: pathway involving downregulated genes in YRT vs. YRC (pink), ORT vs. ORC (light blue), or both comparisons (pink and light blue). The edge indicates gene overlap between pathways; edge thickness represents Jaccard Overlap combined coefficient. Pathway details in Datasets S3 and S4.
(F) Dot plots of the gene expression of TGF-β signaling pathways and ECM organization pathways in YRT vs. YRC (red) and ORT vs. ORC (blue) comparisons. Color gradient: scaled -log_{10}(p-value).

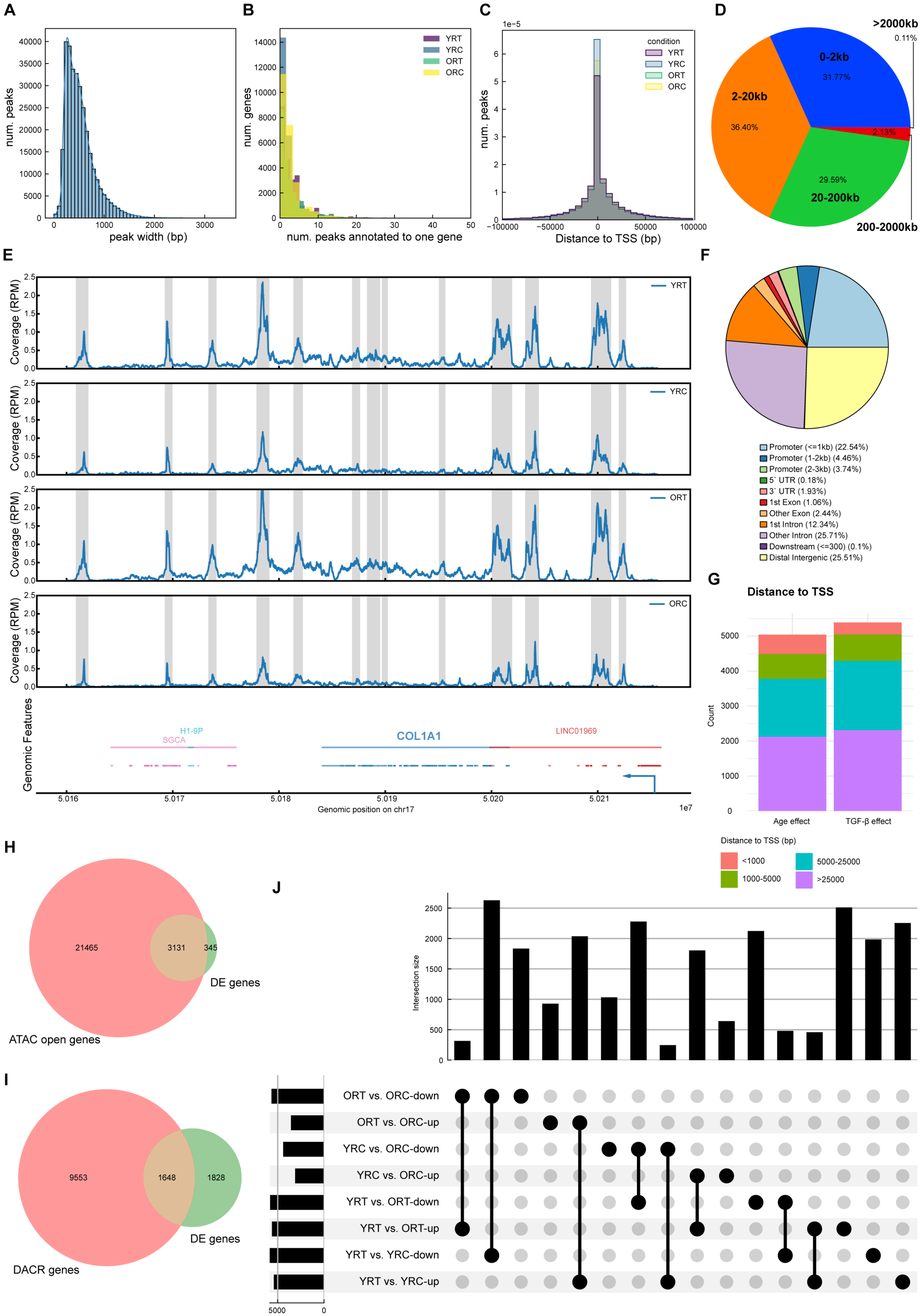
A
B
C
D
num. peaks
peak width (bp)
YRT
YRC
ORT
ORC
num. genes
num. peaks annotated to one gene
condition
Distance to TSS (bp)
>2000kb
0.11%
0-2kb
31.77%
2-20kb
36.40%
2.13%
29.59%
20-200kb
200-2000kb
E
F
Coverage (RPM)
Promoter (<=1kb) (22.54%)
Promoter (1-2kb) (4.46%)
Promoter (2-3kb) (3.74%)
5` UTR (0.18%)
3` UTR (1.93%)
1st Exon (1.06%)
Other Exon (2.44%)
1st Intron (12.34%)
Other Intron (25.71%)
Downstream (<=300) (0.1%)
Distal Intergenic (25.51%)
G
Distance to TSS
Genomic Features
H1-9P
SGCA
COL1A1
LINC01969
Genomic position on chr17
Count
Age effect
TGF-β effect
Distance to TSS (bp)
<1000
1000-5000
5000-25000
>25000
H
21465
3131
345
DE genes
ATAC open genes
J
Intersection size
I
9553
1648
1828
DE genes
DACR genes
ORT vs. ORC-down
ORT vs. ORC-up
YRC vs. ORC-down
YRC vs. ORC-up
YRT vs. ORT-down
YRT vs. ORT-up
YRT vs. YRC-down
YRT vs. YRC-up

Figure S6 ATAC-seq open peaks and DACRs analysis, related to Figure 4
(A) Peak width distribution of ATAC-seq open peaks in YRT, YRC, ORT, and ORC. bp: base pair.
(B) Distribution of the number of open peaks annotated to one gene.
(C) Distribution of the open peaks' distance to TSS.
(D) Pie chart of the fraction of open peaks' distance to TSS.
(E) Enrichment of ATAC-seq signal over background (y axis, log fold) along the COL1A1 (x axis, chr17:50,184,101-50,201,631 loci).
(F-G) The fraction of DACRs (FDR < 0.01, |log2(fold change)| > 1) from 8 pairwise comparisons listed in (J) at **(F)** different annotation locations and **(G)** different distances to the nearest TSS. UTR, untranslated region.
(H-I) Venn diagrams of overlapping genes of **(H)** ATAC-seq open peaks and **(I)** DACRs with DE genes from the same 8 pairwise comparisons.
(J) Upset plot of DACRs in each pairwise comparison. Intersection size threshold 200.

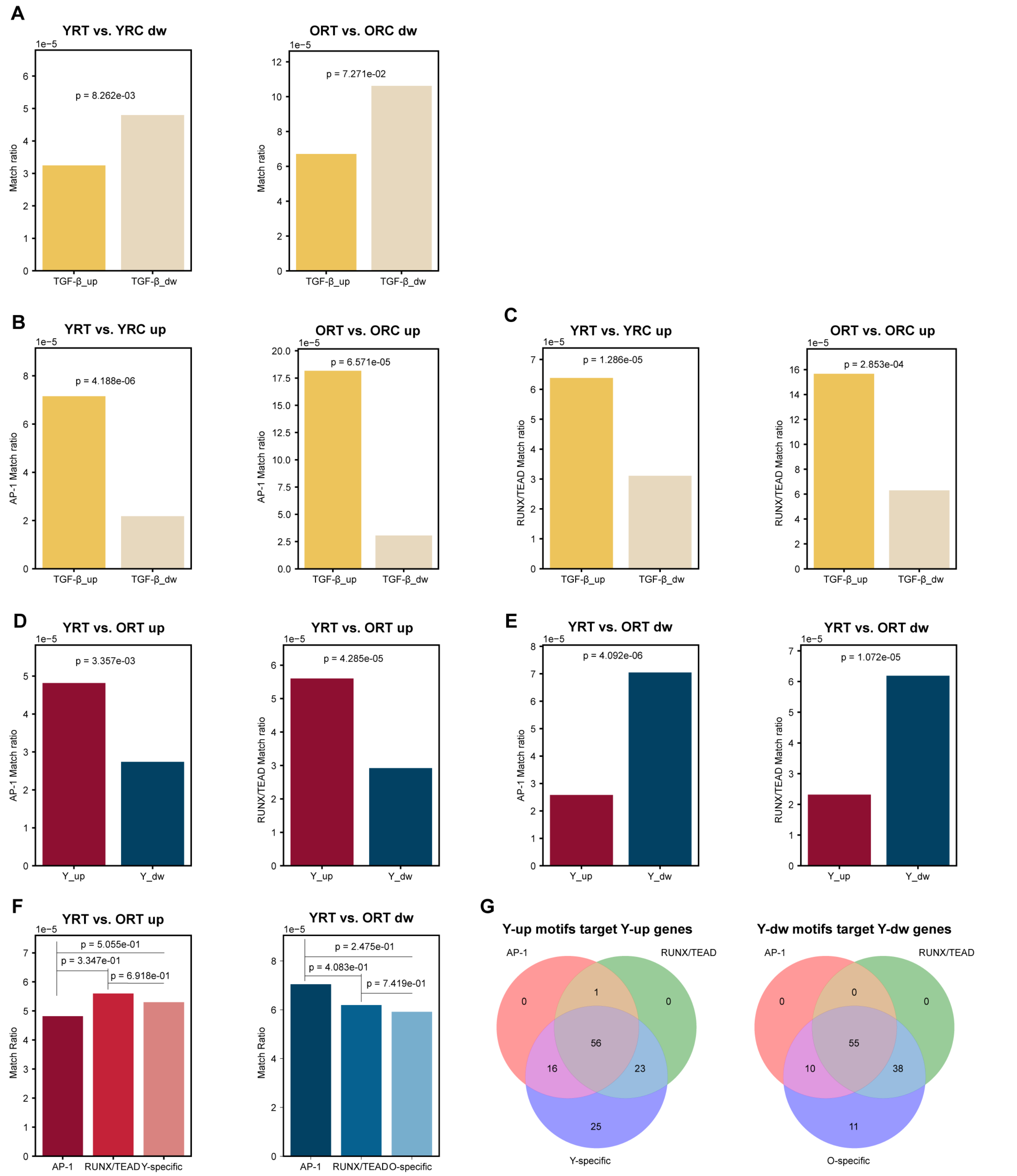

Figure S7 Match ratios of DE genes and enriched motifs in DACRs, related to Figure 4
(A) Bar plots of the downregulated gene and motif match ratio. Motifs enriched in TGF-β_dw DACRs show a greater match ratio with TGF-β-induced downregulated genes, i.e., downregulated genes in YRT vs. YRC (left panel) and ORT vs. ORC (right panel), than motifs in TGF-β_up DACRs.
(B-C) Bar plots of the upregulated gene and **(B)** AP-1 group and **(C)** RUNX/TEAD group motifs enriched in TGF-β effect DACRs match ratio.
(D-E) Bar plots of the **(D)** upregulated gene and **(E)** downregulated and AP-1 group (left panel) or RUNX/TEAD group (right panel) motifs enriched in Age effect DACRs match ratio.

(F) Bar plots of upregulated genes and selected motifs enriched in Y_up (left panel) or downregulated and selected motifs enriched in Y_dw (right panel) DACRs match ratio.
(G) Venn diagrams of selected Y_up DACRs enriching motifs matched upregulated (left panel) or Y_dw DACRs enriching motifs matched downregulated (right panel) genes.
P-values are calculated by the Proportion *Z*-Test.

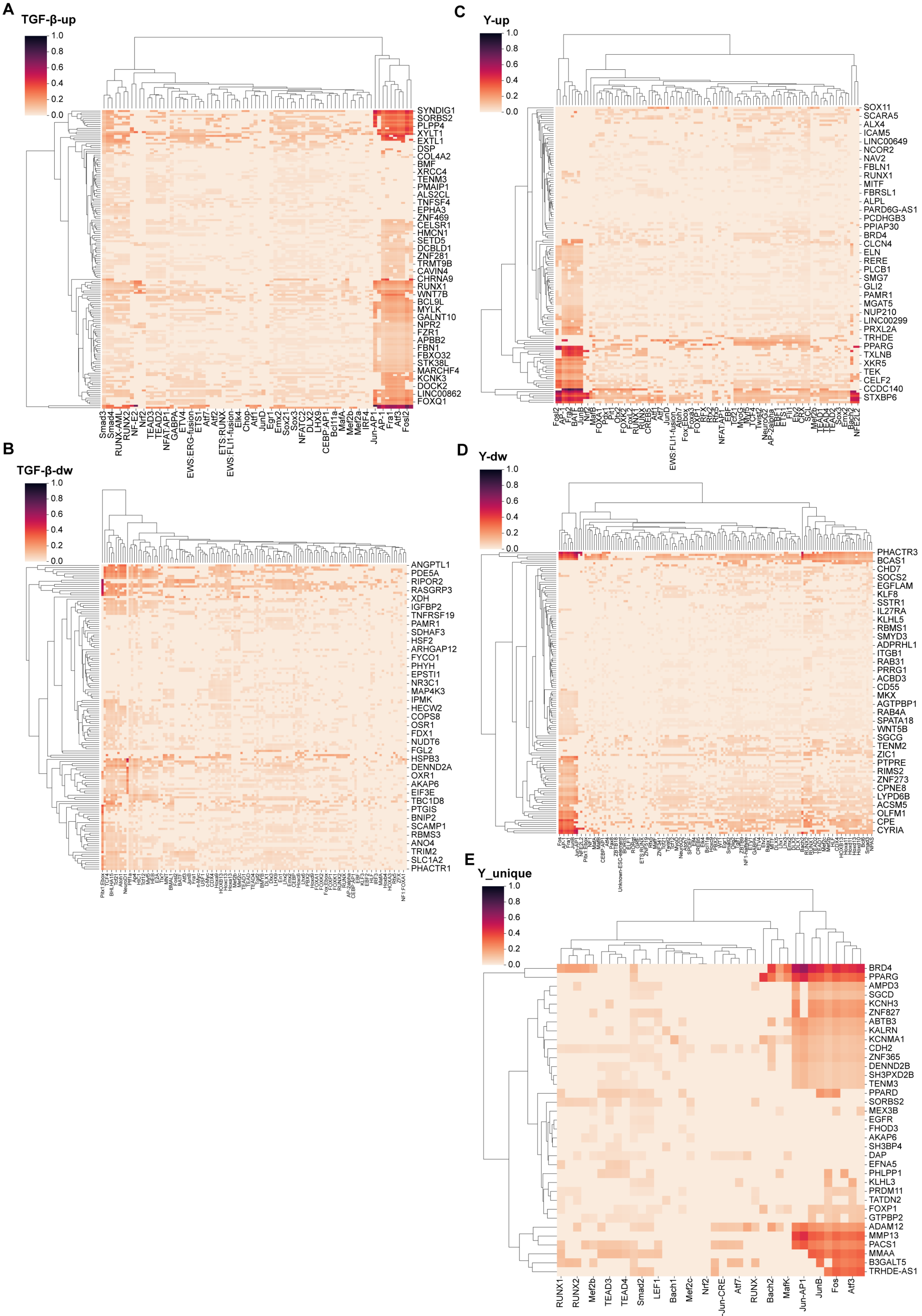
A
TGF-β-up
1.0
0.8
0.6
0.4
0.2
0.0
SYNDIG1
SORBS2
PLPP4
XYLT1
EXTL1
DSP
COL4A2
BMF
XRCC4
TENM3
PMAIP1
ALS2CL
TNFSF4
EPHA3
ZNF469
CELSR1
HMCN1
SETD5
DCBLD1
ZNF281
TRMT9B
CAVIN4
CHRNA9
RUNX1
WNT7B
BCL9L
MYLK
GALNT10
NPR2
FZR1
APBB2
FBN1
FBXO32
STK38L
MARCHF4
KCNK3
DOCK2
LINC00862
FOXQ1
Smad3
Smad4
RUNX-AML
RUNX2
NF-E2
Nrf2
TEAD3
TEAD2
NFAT:AP1
GABPA
ETV4
EWS:ERG-fusion
ETS1
Atf7
Atf2
ETS:RUNX
EWS:FLI1-fusion
Elk4
Chop
Atf1
JunD
Egr1
Emx2
Sox21
Sox3
NFATC2
DLX2
LHX9
CEBP:AP1
Bcl11a
MafA
Mef2b
Mef2a
IRF4
Jun-AP1
AP-1
Fra1
Atf3
Fosl2
B
TGF-β-dw
ANGPTL1
PDE5A
RIPOR2
RASGRP3
XDH
IGFBP2
TNFRSF19
PAMR1
SDHAF3
HSF2
ARHGAP12
FYCO1
PHYH
EPSTI1
NR3C1
MAP4K3
IPMK
HECW2
COPS8
OSR1
FDX1
NUDT6
FGL2
HSPB3
DENND2A
OXR1
AKAP6
EIF3E
TBC1D8
PTGIS
BNIP2
SCAMP1
RBMS3
ANO4
TRIM2
SLC1A2
PHACTR1
C
Y-up
SOX11
SCARA5
ALX4
ICAM5
LINC00649
NCOR2
NAV2
FBLN1
RUNX1
MITF
FBRSL1
ALPL
PARD6G-AS1
PCDHGB3
PPIAP30
BRD4
CLCN4
ELN
RERE
PLCB1
SMG7
GLI2
PAMR1
MGAT5
NUP210
LINC00299
PRXL2A
TRHDE
PPARG
TXLNB
XKR5
TEK
CELF2
CCDC140
STXBP6
D
Y-dw
PHACTR3
BCAS1
CHD7
SOCS2
EGFLAM
KLF8
SSTR1
IL27RA
KLHL5
RBMS1
SMYD3
ADPRHL1
ITGB1
RAB31
PRRG1
ACBD3
CD55
MKX
AGTPBP1
RAB4A
SPATA18
WNT5B
SGCG
TENM2
ZIC1
PTPRE
RIMS2
ZNF273
CPNE8
LYPD6B
ACSM5
OLFM1
CPE
CYRIA
E
Y_unique
BRD4
PPARG
AMPD3
SGCD
KCNH3
ZNF827
ABTB3
KALRN
KCNMA1
CDH2
ZNF365
DENND2B
SH3PXD2B
TENM3
PPARD
SORBS2
MEX3B
EGFR
FHOD3
AKAP6
SH3BP4
DAP
EFNA5
PHLPP1
KLHL3
PRDM11
TATDN2
FOXP1
GTPBP2
ADAM12
MMP13
PACS1
MMAA
B3GALT5
TRHDE-AS1
RUNX1
RUNX2
Mef2b
TEAD3
TEAD4
Smad2
LEF1
Bach1
Mef2c
Nrf2
c-Jun-CRE
Atf7
RUNX
Bach2
MafK
Jun-AP1
JunB
Fos
Atf3

Figure S8 Complete motif-gene regulation weight score heatmaps of TGF-β- and Age-effect DACRs, related to Figure 5
(A-E) Motif-gene regulation weight score heatmaps of **(A)** TGF-β-up, **(B)** TGF-β-dw, **(C)** Y-up, **(D)** Y-dw, and **(E)** Y_unique effect DACRs.

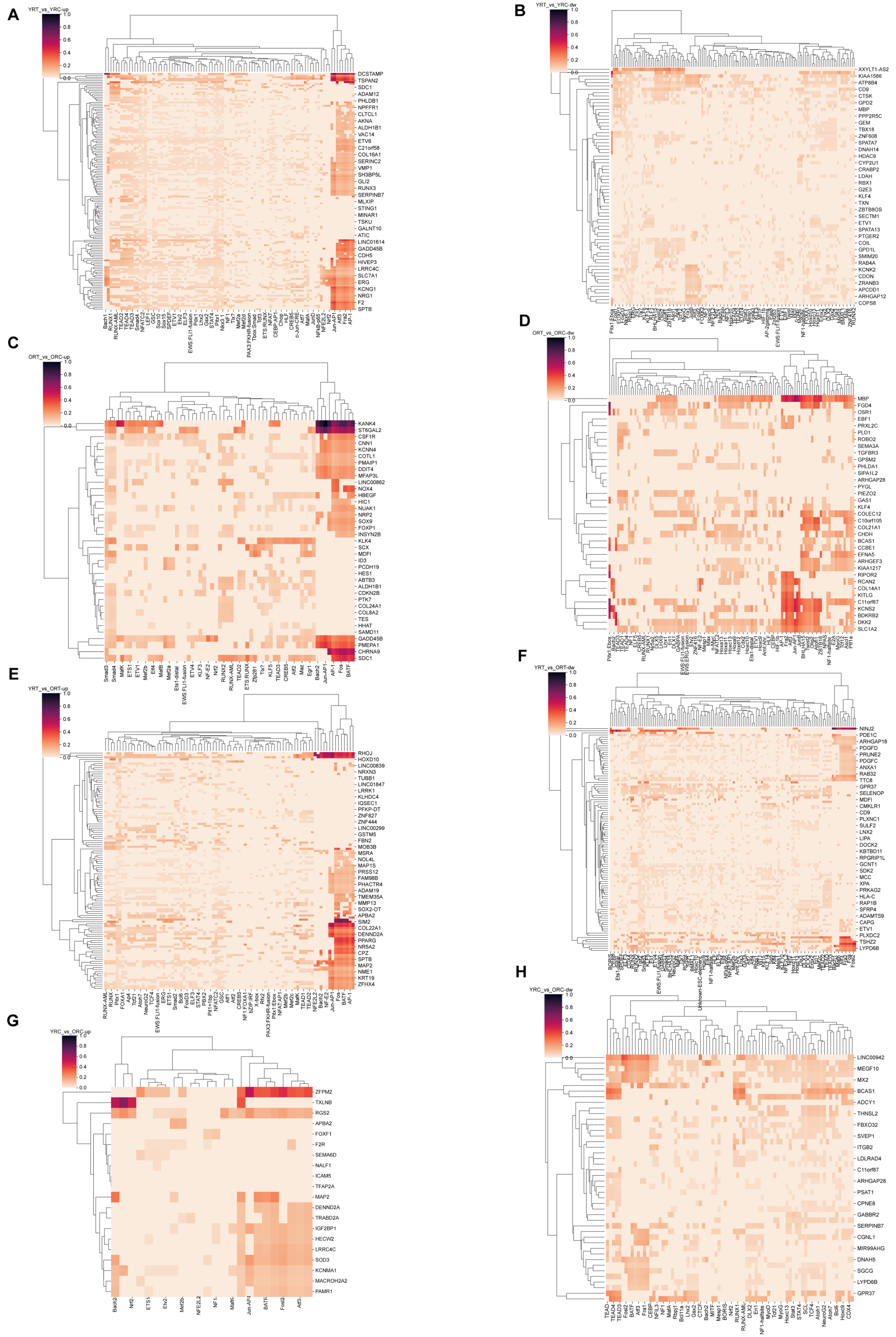
A
YRT_vs_YRC-up
B
YRT_vs_YRC-dw
C
ORT_vs_ORC-up
D
ORT_vs_ORC-dw
E
YRT_vs_ORT-up
F
YRT_vs_ORT-dw
G
YRC_vs_ORC-up
H
YRC_vs_ORC-dw

Figure S9 Complete motif-gene regulation weight score heatmaps, related to Figure 5
(A-H) Motif-gene regulation weight score heatmaps of motifs enriched in DACRs and DE genes in the indicated pairwise comparison.

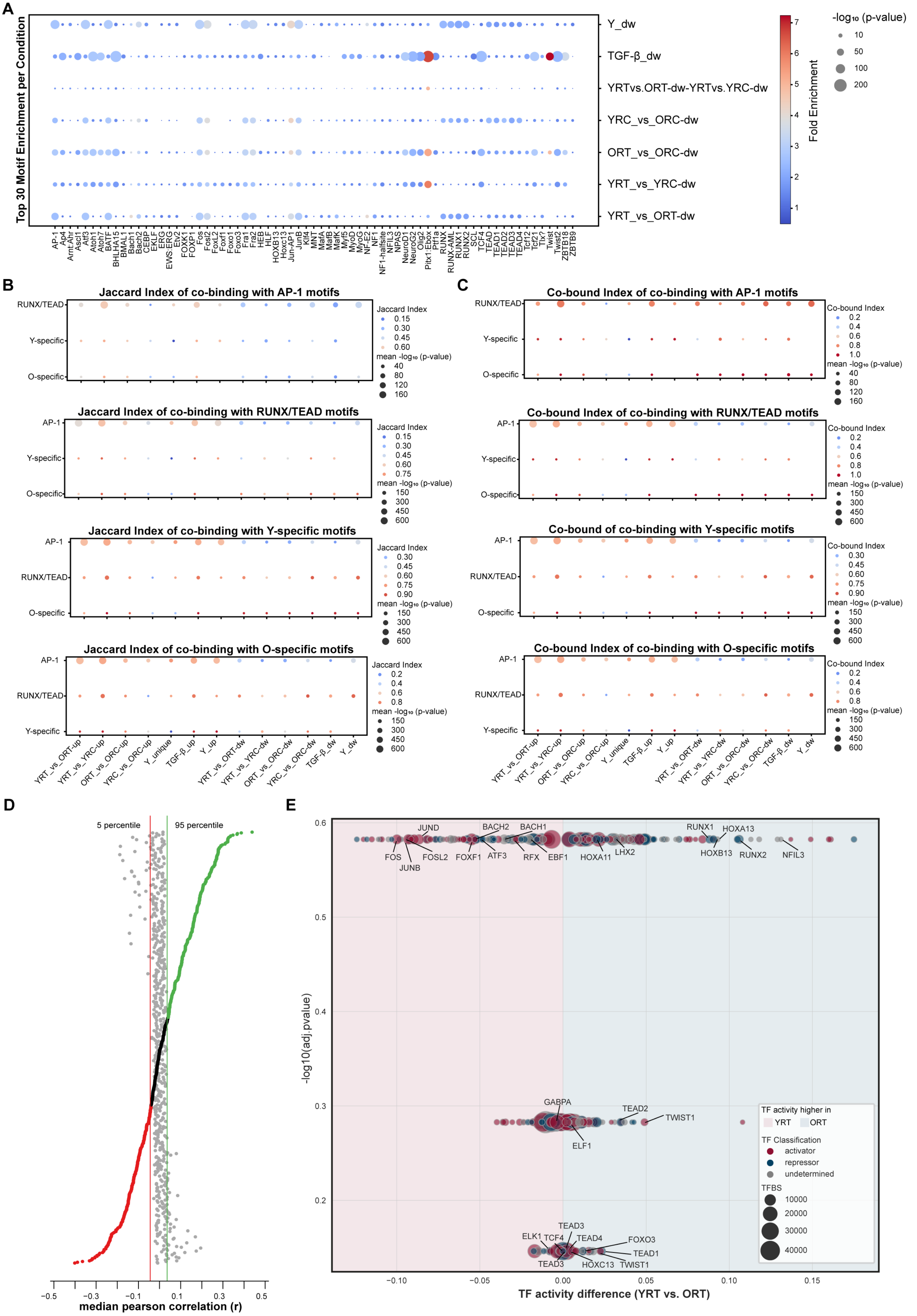
A
Top 30 Motif Enrichment per Condition
Y_dw
TGF-β_dw
YRTvs.ORT-dw-YRTvs.YRC-dw
YRC_vs_ORC-dw
ORT_vs_ORC-dw
YRT_vs_YRC-dw
YRT_vs_ORT-dw
Fold Enrichment
-log₁₀ (p-value)
10
50
100
200
AP-1
Ap4
Arnt:Ahr
Ascl1
Atf3
Atoh1
Atoh7
BATF
BHLHA15
BMAL1
Bach1
Bach2
CEBP
EKLF
ERG
EWS:ERG
Etv2
FOXK1
FOXP1
Fos
Fosl2
FoxL2
Foxf1
Foxo1
Foxo3
Fra1
Fra2
HEB
HLF
HOXB13
Hoxc13
Jun-AP1
JunB
Klf4
MNT
MafA
MafB
MafK
Myf5
MyoD
MyoG
NF-E2
NF1
NF1-halfsite
NFIL3
NPAS
NeuroD1
NeuroG2
Olig2
Pitx1:Ebox
Ptf1a
RUNX
RUNX-AML
RUNX1
RUNX2
SCL
TCF4
TEAD
TEAD1
TEAD2
TEAD3
TEAD4
Tcf12
Tcf21
Tlx?
Twist
Twist2
ZBTB18
ZBTB9
B
Jaccard Index of co-binding with AP-1 motifs
Jaccard Index of co-binding with RUNX/TEAD motifs
Jaccard Index of co-binding with Y-specific motifs
Jaccard Index of co-binding with O-specific motifs
RUNX/TEAD
AP-1
Y-specific
O-specific
Jaccard Index
mean -log₁₀ (p-value)
C
Co-bound Index of co-binding with AP-1 motifs
Co-bound Index of co-binding with RUNX/TEAD motifs
Co-bound of co-binding with Y-specific motifs
Co-bound Index of co-binding with O-specific motifs
Co-bound Index
YRT_vs_ORT-up
YRT_vs_YRC-up
ORT_vs_ORC-up
YRC_vs_ORC-up
Y_unique
TGF-β_up
Y_up
YRT_vs_ORT-dw
YRT_vs_YRC-dw
ORT_vs_ORC-dw
YRC_vs_ORC-dw
TGF-β_dw
Y_dw
D
5 percentile
95 percentile
median pearson correlation (r)
E
JUND
BACH2
BACH1
RUNX1
HOXA13
FOS
JUNB
FOSL2
FOXF1
ATF3
RFX
EBF1
HOXA11
LHX2
HOXB13
RUNX2
NFIL3
GABPA
TEAD2
TWIST1
ELF1
TEAD3
ELK1
TCF4
TEAD4
FOXO3
TEAD1
HOXC13
-log10(adj.pvalue)
TF activity difference (YRT vs. ORT)
TF activity higher in
YRT
ORT
TF Classification
activator
repressor
undetermined
TFBS
10000
20000
30000
40000

Figure S10 Motifs enrichment, cooccupancy, classification, and activities, related to Figure 5
(A) Enriched motifs in less accessible DACRs. The top 30 motifs in each category of DACRs are shown with the dot size indicating $-\log_{10}$(p-value) and the dot color indicating Fold Enrichment.
(B) Jaccard index calculated by the number of occupied peaks divided by the sum of peak numbers between the two groups. Dot color: index values; dot size: mean motif enrichment $-\log_{10}$(p-value) of group motifs.
(C) Co-bound index calculated by the number of occupied peaks divided by the number of peaks in the reference group. Dot color: index values; dot size: mean motif enrichment $-\log_{10}$(p-value) of group motifs.
(D) TF median correlations are ordered from positive (top) to negative (bottom). TFs were classified as activator (green) or repressor (red) when the correlation with their putative binding sites was outside the 95th (green vertical line) and 5th (red vertical line) percentiles from the distribution of all correlations at non-putative TFBS, respectively.
(E) Volcano plot of differential TF activities between YRT and ORT (x-axis). Selected TFs that are in AP-1, RUNX/ TEAD, Y-specific, and O-specific groups are labeled. P-values are adjusted by the Benjamini-Hochberg procedure and obtained through diffTF (y-axis).

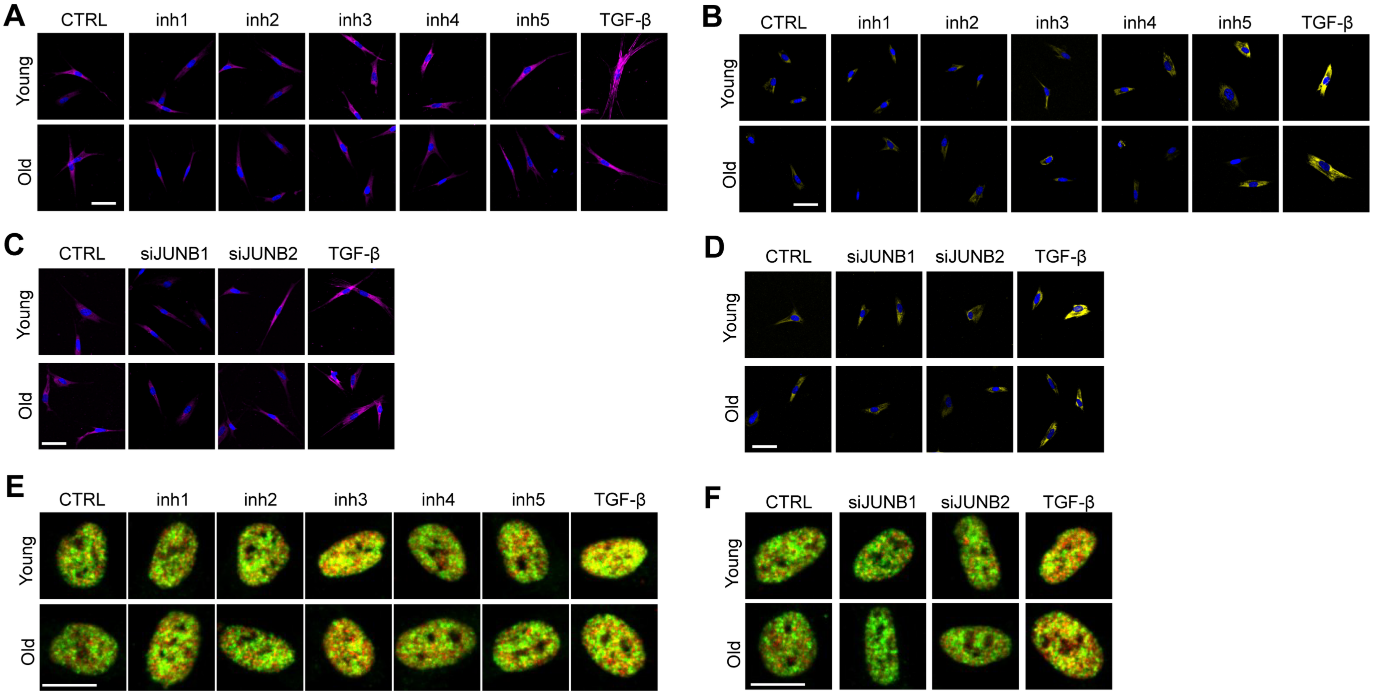

Figure S11 Validation experiments using siRNA and inhibitors of AP-1, related to Figure 6
(A-B) Representative images of normalized expression values of protein markers, **(A)** aSMA and **(B)** COL1A1, of fibroblasts in tensed matrix with or without inhibitor treatments. CTRL: +DMSO/-TGF-β; inh1:+ LY294002/+TGF-β; inh2: +SB203580/+TGF-β; inh3: +PD98059/+TGF-β; inh4: +SP600125/+TGF-β; inh5: +T5224/+TGF-β; TGF-β: +DMSO/+TGF-β. Scale bar: 50μm.
(C-D) Representative images of normalized expression values of protein markers, **(C)** aSMA and **(D)** COL1A1, of fibroblasts in tensed matrix with or without siRNA transfection. CTRL: +siNC/-TGF-β; siJUNB11:+ siJUNB1/+TGF-β; siJUNB2: +siJUNB2/+TGF-β; TGF-β: +siNC/+TGF-β (siNC: Negative Control of siRNA). Scale bar: 50μm.
(E-F) Representative images of colocalization of JUNB with RNA polymerase **II**. Volume ratio: overlapped volume of TF and RNA polymerase **II** protein normalized by nuclear volume. **(E)** fibroblasts with or without inhibitor treatments; **(F)** fibroblasts with or without siRNA transfection. Scale bar: 10μm.

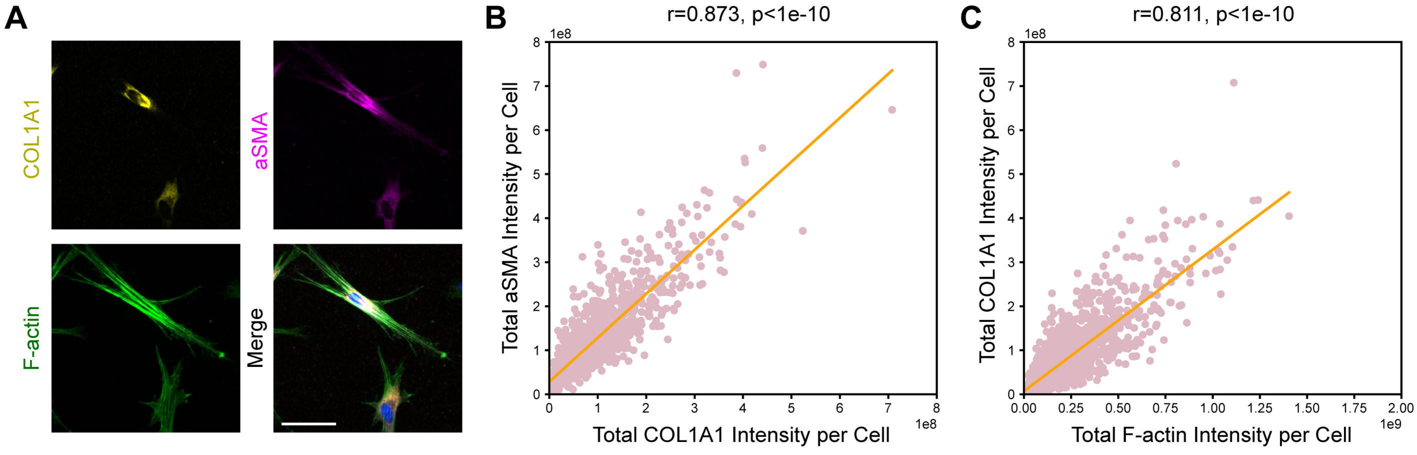

Figure S12 Correlation of protein expression at the single cell level, related to Figure 2

(A) Representative images of COL1A1, aSMA, and F-actin co-stained young fibroblasts. Scale bar: 50μm.

(B) Scatter plot of expression values of protein markers per cell. X-axis: total intensity of COL1A1 per cell; y-axis: total intensity of aSMA per cell.

(C) Scatter plot of expression values of protein markers per cell. X-axis: total intensity of F-actin per cell; y-axis: total intensity of COL1A1 per cell.

Datasets (separate file)

Dataset S1. Excel file containing enriched pathways of upregulated genes in YRT vs. YRC, related to Figure 3D and Figure S5D

Dataset S2. Excel file containing enriched pathways of upregulated genes in ORT vs. ORC, related to Figure 3D and Figure S5D

Dataset S3. Excel file containing enriched pathways of downregulated genes in YRT vs. YRC, related to Figure S5E

Dataset S4. Excel file containing enriched pathways of downregulated genes in ORT vs. ORC, related to Figure S5E

Dataset S5. TFs selected as AP-1, RUNX/TEAD, Y_specific, and O_specific TF groups, related to Figure S7 and Figure 5

SI Reference

1. J. Reimand, *et al.*, Pathway enrichment analysis and visualization of omics data using g:Profiler, GSEA, Cytoscape and EnrichmentMap. *Nat. Protoc.* **14**, 482–517 (2019).

2. M. Hatakeyama, *et al.*, SUSHI: an exquisite recipe for fully documented, reproducible and reusable NGS data analysis. *BMC Bioinformatics* **17**, 228 (2016).

3. I. Berest, *et al.*, Quantification of Differential Transcription Factor Activity and Multiomics-Based Classification into Activators and Repressors: diffTF. *Cell Rep.* **29**, 3147-3159.e12 (2019).

4. S. Venkatachalapathy, D. S. Jokhun, G. V. Shivashankar, Multivariate analysis reveals activation-primed fibroblast geometric states in engineered 3D tumor microenvironments. *Mol. Biol. Cell* **31**, 803–812 (2020).
